# The effects of *Saccharomyces cerevisiae* strains carrying alcoholic fermentation on the fermentative and varietal aroma profiles of young and aged Tempranillo wines

**DOI:** 10.1016/j.fochx.2021.100116

**Published:** 2021-02-09

**Authors:** Marie Denat, Dolores Pérez, José María Heras, Amparo Querol, Vicente Ferreira

**Affiliations:** a Laboratory for Aroma Analysis and Enology (LAAE), Department of Analytical Chemistry, Universidad de Zaragoza, Instituto Agroalimentario de Aragón (IA2) (UNIZAR-CITA), c/Pedro Cerbuna 12, 50009 Zaragoza, Spain; b Lallemand Bio S.L., 08028 Barcelona, Spain; c Institute of Agrochemistry and Food Technology (IATA-CSIC), 46980 Paterna, Valencia, Spain; d Estación Experimental Agropecuaria Mendoza (EEA), Instituto Nacional de Tecnología Agropecuaria (INTA), 5507 Luján de Cuyo, Mendoza, Argentina

**Keywords:** Fermentation, Longevity, Fruity esters, Volatile phenols, Norisoprenoids, Vanillin, Strecker aldehydes, Ethyl leucate

## Abstract

Ten different *Saccharomyces cerevisiae* strains fermented semi-synthetic musts containing a Polyphenolic and Aroma Precursor Fraction (PAF) extracted from Tempranillo grapes. Aroma compounds were studied by Gas Chromatography (GC), GC-Olfactometry and GC-Mass Spectrometry (MS), during fermentation by trapping volatilized aroma, immediately after fermentation and after accelerated aging. Volatiles lost by evaporation during fermentation are mostly fermentative compounds and not grape-related odorants. Isobutanal and some esters are mostly lost during fermentation. In many cases the impact of yeast strain is evident only after aging. Strains could be classified into 3 major clusters with marked differences in fermentative and varietal profiles. Linalool and geraniol were found to have fermentative origin. *S. cerevisiae* yeast strains can effectively modulate varietal aroma, likely through specific enzymatic activities acting on grape phenolic acids and norisoprenoid aroma precursors and may be specifically used to mitigate some aging-related off odours, such as massoia lactone, guaiacol or TDN.

## Highlights

• 10 *Saccharomyces cerevisiae* strains fermented must with phenolics and aroma precursors.
• Isobutanal, isopropyl isoamyl acetates, and ethyl propanoate lost by evaporation.
• Yeast strain affects levels of 45 out of 60 aroma compounds mostly after aging.
• Linalool and geraniol fermentative aroma compounds.
• Strong modulation of varietal aroma. Strains can limit levels of guaiacol or TDN.

## 1 Introduction

Wine aroma is its most outstanding sensory property and is essential for its quality and differentiation (Charters & Pettigrew, 2007). Although the number of volatile molecules which can be a part of the volatile fraction of wines is very large, exceeding most likely several thousands, it has been suggested that 70 different odour chemicals are those playing major roles on the aromatic properties of wines (De-la-Fuente-Blanco, Sáenz-Navajas, & Ferreira, 2020).

Quantitatively, the most abundant wine odorants are alcoholic fermentation (AF) by-products, particularly higher alcohols, ethyl esters and acetates, some carbonyls and acids. By contrast, many other relevant odorants derive from grape specific precursors and can be present at very limited concentrations, within the ng/L range in the case of polyfunctional mercaptans, few hundreds of ng/L in the case of β-ionone, around the few μg/L in the case of β-damascenone or below 0.2 mg/L in the cases of terpenols, volatile phenols and vanillin derivatives (Ferreira & Lopez, 2019, Ruiz et al., 2019).

There are also relevant differences between compounds regarding the time at which they are formed or liberated. Most fermentation by-products derive from yeast amino acid and fatty acid metabolisms (Swiegers, Bartowsky, Henschke, & Pretorius, 2005), so that they are significantly formed from the early stages of AF and are known to be partly lost by evaporation (Gómez-Plaza et al., 1993, Guerrini et al., 2016). Some other compounds, such as the monoterpenoids geraniol and linalool, are directly liberated from grape-specific glycosylated precursors during fermentation by the action of yeast β-glucosidases (Gunata, Bitteur, Brillouet, Bayonove, & Cordonnier, 1988). Polyfunctional mercaptans are also byproducts of AF and may be also partially lost by evaporation during fermentation.

On the contrary, the formation and accumulation of some other volatiles requires aging time. In fact, a quantitatively relevant group of aroma compounds and aroma precursors are subjected to several slow chemical reactions such as acid hydrolysis, esterification or intra-molecular rearrangements that will greatly affect wine aroma profile. This is the case of most norisoprenoids and vanillin derivatives, such as β-damascenone and TDN or vanillin and acetovanillone, whose levels increase during aging as the consequence of different transformations of grape carotenoid metabolites (Winterhalter & Gök, 2013) or of grape glycosylated precursors (Ferreira & Lopez, 2019). Notably, it has been recently observed that the yeast genera plays a major role in the modulation of TDN concentration in wine after some time of aging (Oliveira & Ferreira, 2019). A third group of odorants suffering changes during aging are labile molecules, such as linalool or geraniol, which degrade during aging to form α-terpineol, nerol or 1,8-cineole (Waterhouse, Sacks, & Jeffery, 2016). Other groups of odorants deeply affected by aging are fruity esters and acetates. The acetates of higher alcohols are quickly hydrolysed and their levels soon fade away. On the contrary, the ethyl esters of branched acids - isobutyric, 2-methylbutyric and isovaleric acids - and of other minor acids, slowly and steadily increase by esterification of their precursor acids with ethanol (Díaz-Maroto, Schneider, & Baumes, 2005). The existence of all these processes makes that aging time should be considered as an important factor to assess the role of yeasts on wine aroma modulation.

*Saccharomyces cerevisiae* is the micro-organism most widely studied regarding its sensory impact on wine (Tempère et al., 2018). However, most previous studies have dealt with the short-term impact of this yeast on both fermentative and varietal aroma profiles (Gamero et al., 2011, Gammacurta et al., 2017, Molina et al., 2009), neglecting aging effects and also possible differences in the aroma profiles lost by evaporation, which could be particularly relevant in lab-scale fermentations. Both aspects will be specially addressed in the present work whose objective is to evaluate the impact of *S. cerevisiae* yeast strains on the fermentative and varietal aroma of Tempranillo wine during and after fermentation, and after a period of accelerated aging.

## 2 Material and methods

### 2.1 Reagents and standards

Dichloromethane and methanol (≥99%) Disto-Pesticide residue grade were supplied by Merck (Darmstadt, Germany). ACS quality absolute ethanol was purchased from Panreac (Barcelona, Spain). 2-Butanol (≥99%), 4-methyl-2-pentanol (99%), 4-hydroxy-4-methyl-2-pentanone (99%), ethyl heptanoate (99%) and heptanoic acid (99%) were used as internal standards for major compounds analysis. 2-Octanol (99.5%), 3-octanone (99%) and 3,4-dimethylphenol (99%) were used as internal standards for minor compounds analysis. They were purchased from Merck as well as the chemical standards used in this study (>98%), except for TDN which was synthesized by Synchem UG &Co (Felsberg, Germany) with a purity of 80%. LiChrolut EN resin cartridges were obtained from Merck (Darmstadt, Germany). Sep Pak-C18 resins, prepacked in 10 g cartridges were from Waters (Ireland).

### 2.2 Semi-synthetic must preparation

#### 2.2.1 Precursors and phenols extraction from Tempranillo grapes

Phenolic and Aroma precursor Fraction (PAF) was extracted from Tempranillo grapes mistelle prepared at the ICVV of Logroño (Spain) following the procedure developed by Alegre et al. (2020).

#### 2.2.2 Synthetic grape must

Synthetic must was prepared as described in Hernández-Orte, Bely, Cacho, and Ferreira (2006). Nitrogen content was adjusted by mixing 220 mg/L of (NH_4_)_2_HPO_4_, and a mixture of amino acids resembling the average profile of Tempranillo grape variety (Hernández-Orte, Cacho, & Ferreira, 2002). Synthetic glutathionylated and cysteinilated (Cys) precursors of 3-mercaptohexanol (MH) and 4-mercaptopentan-2-one (MMP) were added from a solution in MilliQ water, (0.1 mg/L Cys-MH, 0.05 mg/L Cys-MMP, 1 mg/L Glu-MH, 0.05 mg/L Glu-MMP). After pH adjustment to 3.5 with NaOH, synthetic must was sterilized by filtration (0.45 μm) inside a vertical laminar flow chamber. PAF was dealcoholized, resuspended in sterile distilled water and added to synthetic must at 10% (v/v).

### 2.3 Wine elaboration

#### 2.3.1 Yeasts strains, pre-culture conditions and yeast growth monitoring

Ten *S. cerevisiae* strains (Lallemand Bio SL, Madrid, Spain) were used: Lalvin ICV D254™ (D254), Lalvin Clos™ (CLOS), Uvaferm HPS™ (HPS), Enoferm BDX™ (BDX), Lalvin Rhône2056® (RHONE), Lalvin ICV D80™ (D80), Lalvin 71B™ (71B), Lalvin Persy™ (PERSY), Lalvin ICV OKAY™ (OKAY), IONYS wf™ (IONYS). Active dry yeasts were rehydrated in distilled water at 37 °C for 20 min under agitation, 100 μL of these cultures were plated on Glucose Peptone Yeast (GPY) and incubated at 25 °C for 24 h. Pre-cultures were prepared by inoculating several colonies into 5 mL of GPY broth at 25 °C for 24 h. After quick centrifugation, GPY broth was discarded and colonies were resuspended in distilled water. Living cells were quantified by flow cytometry and each fermenter of synthetic must was inoculated at 10^6^ living cells/mL. Cell viability and vitality were monitored by flow cytometry (Tilloy, Ortiz-Julien, & Dequin, 2014).

#### 2.3.2 Experimental design

All the fermentations were realized in triplicates. Fermentation with CLOS and IONYS were repeated in must without PAF addition, whose volume was replaced by sterile distilled water. Unfermented control of synthetic must with and without PAF was also included in duplicates. Experimental procedure is summarized in Fig. S1.

#### 2.3.3 Fermentation system

Fermentations were carried out in 100 mL glass fermenters containing 50 mL of synthetic must so that headspace represented 50% of the total volume. Fermenters were tightly closed with a perforated silicone cork in which an airlock (Micromalta S.L., Madrid, Spain) was inserted. Needles and 5 mL syringes were inserted into the silicon cork to allow sampling without opening the fermenter.

#### 2.3.4 Fermentation monitoring

Fermentations were carried out at 25 °C, under agitation at 150 rpm using a magnetic stirrer. CO_2_ release was monitored by weighing. The main chemical fermentative parameters were analysed at the beginning and at the end of fermentation, including sugars, acids and alcohols by UHPLC as described in Su et al. (2019).

#### 2.3.5 End of fermentation and accelerated aging

At the end of fermentation, samples were centrifuged and conditioned for accelerated anoxic aging into a free-O_2_ chamber Jacomex (Dagneux, France). Samples were placed into 18 mL glass tubes capped with non-metallic screw caps and bagged in high density plastic bags containing oxygen scavengers AnaeroGenTM (Thermo Scientific, USA) and incubated at 50 °C for 5 weeks. This methodology was developed by Vela, Hernández-Orte, Franco-Luesma, & Ferreira, (2017) and was successfully applied to the aging of fermented media (Oliveira & Ferreira, 2019). It was observed that 5 weeks of anoxic aging at 50 °C were roughly equivalent to one year of aging at room temperature.

### 2.4 Analysis of the aroma evaporated during fermentation

A pre-purified standard SPE cartridge filled with 160 mg LiChrolut-EN resin was lodged into the airlock of the fermenters to trap volatiles emited during fermentation. When this finished, the cartridge was removed, dried and eluted with 1.6 mL of dichloromethane containing 5% (*v/v*) of methanol.

For GC-Olfactometry (GC-O), a single extract was prepared by mixing 100-uL of each one of the forty extracts obtained from the 40 different fermentations. The extract was carefully concentrated by evaporation of the solvent under nitrogen flow until a 0.2 mL final volume and injected into the GC-O system, as described in San-Juan, Pet’ka, Cacho, Ferreira, & Escudero, (2010). Olfactometric scores were obtained by combining intensity and frequency as described by Dravnieks, (1985). The odorants were identified by comparison of their descriptors, chromatographic retention index in DB-WAX and DB-5 columns and mass spectra with those of pure reference compounds.

For quantification of the odorants present in the extracts, these were spiked with the internal standards, concentrated by evaporation under nitrogen up to 0.2 mL and analyzed. Major fermentative compounds were directly quantified by GC-FID analysis under the conditions described in Ortega, López, Cacho, & Ferreira, (2001). Minor compounds were quantified by GC–MS analysis of the extracts in a Shimadzu QP2010 (Quioto, Japan) equipped with a DB-WAXetr GC-column from Agilent (Santa Clara CA, USA), 30 m × 0.25 nm, 0.5 μm of film thickness, preceded by a 2 m × 0.25 mm uncoated pre-column. Carrier gas was Helium at 1.26 mL/min. Injection volume was 1 μL in split mode (ratio 1/30). Injector temperature was 250 °C. Chromatographic oven temperature was initially at 30 °C for 1 min, then raised at 1 °C/min to 35 °C, held for 1 min, then at 1 °C/min to 40 °C, at 15 °C/min to 55 °C, held for 5 min, at 15 °C/min to 72 °C, held for 5 min, at 15 °C/min to 150 °C, held for 15 min. The Ion source was kept at 220 °C and the interface at 230 °C. The mass analyser was set in single ion monitoring mode. The complete list compounds quantified in both procedures, including the *m*/*z* ratios selected for MS quantitation is available in Table S1.

### 2.5 Analysis of major and minor volatile compounds

Major metabolites of alcoholic fermentation (higher alcohols and their acetates, volatile fatty acids and their ethyl esters, branched fatty acids and their ethyl esters, acetoin, diacetyl, and acetaldehyde), usually present in wines at levels above 0.2 mg/L, were analysed by the GC-FID analysis of a dichloromethane microextract as described by Ortega, López, Cacho, & Ferreira, (2001).

Minor aroma compounds present in wine at levels 0.1–200 μg/L (branched ethyl esters, terpenes, norisoprenoids, vanillin derivatives, volatile phenols) were analysed by GC–MS analysis of a SPE extract following the protocol described by López, Aznar, Cacho, & Ferreira, (2002). The complete list of quantified is available in Table S1.

### 2.6 Statistical analysis

Statistical analysis and graphics were realized using R software (RStudio Team, 2020). Significance was determined by analysis of variance (ANOVA) using *anova* function from *car* package (v.3.0.2) and Tuckey’s honestly significance difference test was performed on ANOVA results, using *HSD.test* function from *agricolae* package (v.1.3.1). Principal Component Analysis (PCA) were performed and plotted using *factoextra* package (v.1.0.5).

## 3 Results

### 3.1 Odorants lost during fermentation

In order to assess the type and amounts of odorants lost during fermentation, a small trap was installed in the fermenters. A GC-O screening procedure was carried out on an extract obtained by mixing small aliquots of all the extracts obtained in the experiment. Results are summarized in Table 1. Overall, twenty-two odorants were detected with GC-O scores above 20%. Eleven out of the twenty-two odorants were identified at maxima level of confidence. In five other cases, some of the identity criteria could not be completely fulfilled because of different reasons, such as excessively low levels to get a good MS spectrum (Z and E-2-nonenals), co-elution in the non-polar column (cresol and guaiacol) or discrepancy in the odour (ethyl octanote). The identification of the six less intense odorants was based only on the coincidence of the odour and RI of the odorant in the polar column with those of the standard, and should be considered tentative.

**Table 1:** Identification of odorants purged out during fermentations and trapped in LiChrolut-EN cartridges placed before the Muller valves of the fermenters. The GC-O experiment was carried out on an extract made by mixing the eluates of the different traps. Retention indexes (RI) in DB-WAX and DB-5 columns, identifications, olfactometric scores (MF%) and odor descriptors are presented in the table. Compounds are marked by letters according to the reliability of their identification (see legend).

RI (DB-WAX)	RI (DB-5)	Compound*	MF (%)	Odor description
1220	<900	isoamyl alcohol^A^	87	cheese, rancid
940	<900	3-methylbutanal^A^	78	cheese, rancid
935	<900	isopropyl acetate^A^	62	fruity, strawberry
975	<900	ethyl isobutyrate^A^	62	fruity, strawberry
2097		cresol^B^	55	phenolic, leather
1929	1115	2-phenylethanol^A^	53	floral, rose
1039	<900	ethyl butyrate^A^	46	fruity, strawberry
1241	988	ethyl hexanoate^A^	45	fruity
1513	1133	Z-2-nonenal^B^	38	rancid, cucumber
1128	<900	isoamyl acetate^A^	37	banana
1441	1193	ethyl octanoate^B^	35	mushroom, plastic, humidity
1544	1153	E-2-nonenal^B^	29	fat, rancid
1621	1391	ethyl decanoate^A^	29	soap
1696	<900	2 and 3-methylbutyric acid^A^	26	sweat, rancid
1842		guaiacol^B^	26	smoky, burn
966	<900	ethyl propanoate^A^	24	fruity
1425		1-nonen-3-one^C^	24	mushroom, undergrowth
1468		decanal^C^	24	grass, floral
1953		Z-whisky lactone^C^	24	spicy
2169		2-phenoxyethanol^C^	24	rancid, carton
2245		4-vinylguaiacol^C^	24	spicy
2214		sotolon^C^	22	spicy

* A: identification conclusive. Experimental RIs in two columns, odour and MS corresponded to the one obtained with the pure chemical standard; B: identity highly likely, one of the previous criteria (two RIs, odour, MS) failed; C: tentative identification based on RI on a single column, odour and previous literature.

The most intense odorants were mainly by-products of alcoholic fermentation: isoamyl alcohol and 2-phenylethanol, 3-methylbutanal, and a numerous group of esters (isopropyl and isoamyl acetates, and the ethyl esters of isobutyric, butyric, hexanoic, octanoic and decanoic acids). Apart from these, cresol and Z and E-2-nonenals were also between the twelve most intense. The origin of cresol is not clear, but it could be a breakdown product of the polyphenols present in fermentation media; while nonenals are known derivatives of the auto-oxidation of grape fatty acids. Results, therefore, confirm that the most relevant odorants purged out during fermentation are by-products derived from yeast metabolism or grape fatty acid auto-oxidation and not varietal aroma compounds released from specific aroma precursors in grape, such as terpenols, norisoprenoids or polyfunctional mercaptans.

### 3.2 Quantitative assessment of the volatiles lost during fermentation

The amounts of eighteen odorants trapped in the cartridges installed in the PAF-containing fermenters are summarized in Table 2. In general, levels were low and in some cases were affected by high imprecision. The total mass of volatiles trapped in the cartridges ranged from around 1 mg (D80) to around 2 mg (71B). The major volatile was isobutyraldehyde, which accounts for more than 50% of the total volatiles trapped. Four other odorants, isopropyl acetate, isoamyl acetate, isobutanol, and isoamyl alcohol can be also found at levels above 100 μg. The levels of some volatiles released were significantly related to the yeast strain, although in most cases differences were not very high. Major differences correspond to the strain IONYS, whose cartridges contained highest levels of acetates and of some ethyl esters, followed by the strain 71B, with maxima levels of aldehydes.

**Table 2:** Amounts (in μg) of volatile compounds purged out during fermentation and trapped in LiChrolut-EN cartridges placed before the Muller valves of the fermenters containing 50 mL of synthetic must. The first column gives the significance of the factor yeast on the levels of volatiles found in the fermentations realized with PAF addition (pvalues in bold are inferior to 0.05).

	pvalue	MUST	CLOS	IONYS	71B	BDX	D254	D80	HPS	OKAY	PERSY	RHONE
**Acetates**												
propyl acetate	**6.24E-10**	0.10 ± 0.02	0.6 ± 0.2	4.1 ± 0.3	0.9 ± 0.2	0.49 ± 0.06	0.6 ± 0.2	0.5 ± 0.2	0.9 ± 0.2	2.1 ± 0.4	3.7 ± 0.8	0.9 ± 0.2
isopropyl acetate	**5.80E-05**	1 ± 7	135 ± 11	320 ± 43	158 ± 34	135 ± 33	130 ± 17	129 ± 54	162 ± 43	199 ± 18	250 ± 33	171 ± 23
isobutyl acetate	**4.47E-02**	n.d.	0.7 ± 0.1	6 ± 2	2.0 ± 0.7	1.8 ± 0.5	1.2 ± 0.4	1 ± 1	2.2 ± 0.6	1.4 ± 0.4	3.1 ± 0.1	1.1 ± 0.5
isoamyl acetate	**4.70E-04**	15 ± 4	6 ± 3	104 ± 18	23 ± 17	–	17 ± 11	8 ± 15	18.2 ± 8.1	29 ± 5	–	14 ± 31
**Acids**												
3-methylbutyric acid	1.14E-01	n.d.	3 ± 1	4 ± 3	4 ± 2	2.4 ± 0.2	4 ± 1	1.8 ± 0.4	5.1 ± 2.0	2.7 ± 3.0	3 ± 2	4 ± 1
2-methylbutyric acid	7.92E-02	0.1 ± 0.1	0.4 ± 0.2	0.8 ± 0.7	0.9 ± 0.6	0.59 ± 0.09	0.9 ± 0.4	0.41 ± 0.09	1.1 ± 0.5	1.4 ± 0.8	0.6 ± 0.6	1. 0 ± 0.3
**Alcohols**												
isobutanol	**4.69E-02**	74 ± 20	130 ± 24	129 ± 8	128 ± 4	–	176 ± 30	142 ± 112	184 ± 31	127 ± 54	–	120 ± 16
isoamyl alcohol	1.57E-01	387 ± 13	420 ± 107	488 ± 58	484 ± 226	–	497 ± 163	325 ± 318	507 ± 125	664 ± 116	–	364 ± 110
**Carbonyls**												
acetaldehyde	**3.07E-03**	5 ± 2	3 ± 2	3.6 ± 0.6	14 ± 10	–	3.3 ± 0.8	4 ± 1	4 ± 1	4 ± 2	–	5 ± 2
isobutyraldehyde	6.39E-02	n.d.	904 ± 482	684 ± 124	1291 ± 528	957 ± 464	548 ± 268	392 ± 210	692 ± 367	493 ± 267	644 ± 243	534 ± 113
2-methylbutanal	5.85E-02	n.d.	1.4 ± 0.2	2.2 ± 0.7	3 ± 1	1.1 ± 0.5	1 ± 1	0.7 ± 0.1	1.2 ± 0.8	2.4 ± 0.8	1.7 ± 0.1	0.9 ± 0.9
3-methylbutanal	2.05E-01	n.d.	3.5 ± 0.9	7 ± 1	12 ± 4	4.2 ± 2.3	3 ± 4	3. 0 ± 0.3	4 ± 3	10 ± 4	6.5 ± 0.3	4 ± 4
**Esters**												
ethyl propanoate	**5.28E-04**	0.1 ± 0.2	6.5 ± 0.6	38 ± 18	5 ± 1	4.9 ± 0.3	6 ± 7	5 ± 2	7 ± 2	7.5 ± 0.5	10 ± 3	6 ± 2
ethyl butyrate	**2.95E-02**	0.2 ± 0.1	0.10 ± 0.03	0.86 ± 0.05	0.19 ± 0.01	–	0.23 ± 0.04	0.1 ± 0.1	0.3 ± 0.1	0.20 ± 0.03	–	0.2 ± 0.1
ethyl isobutyrate	**3.07E-03**	n.d.	0.3 ± 0.2	0.30 ± 0.05	0.32 ± 0.01	0.34 ± 0.15	0.5 ± 0.2	0.21 ± 0.09	0.5 ± 0.1	0.32 ± 0.06	0.38 ± 0.02	0.4 ± 0.1
ethyl hexanoate	3.09E-01	2.7 ± 0.8	4 ± 2	7.0 ± 0.6	6 ± 5	–	5.9 ± 2.3	3 ± 1	4 ± 2	6.7 ± 0.9	–	3 ± 5
ethyl octanoate	4.89E-01	10 ± 5	7 ± 4	11 ± 2	11 ± 8	–	11 ± 4	5 ± 1	7 ± 3	14 ± 4	–	6 ± 9
ethyl decanoate	**1.23E-04**	0.32 ± 0.05	2.2 ± 0.7	8 ± 2	4 ± 2	–	4 ± 1	2.9 ± 0.9	3.6 ± 0.9	6.3 ± 0.1	–	5 ± 1

n.d. indicates that the compound was not detected or below detection limits.

– indicates that data is not available.

As expected, volatiles purged during fermentation are mostly non-polar aroma compounds such as esters, acetates or carbonyls. Only two acids and two alcohols were found amongst the quantifiable volatile compounds. In these cases, the amounts evaporated corresponded to very small fractions of the volatiles produced. In the particular case of isoamyl alcohol, the 664 μg found in the cartridge of OKAY strain correspond to 13 mg/L in the 50 mL of liquid. Considering that the recently fermented wine contained around 190 mg/L of this compound (Table 2, Supplementary material), it can be estimated that less than 5% of the total amount of isoamyl alcohol produced was evaporated. On the opposite side, isobutyraldehyde was found in the cartridge from the strain 71B at 1.29 mg, which amounts to 26 mg/L in the 50-mL volume, while reported levels of this compound in wine are well below 0.1 mg/L (Culleré, Cacho, & Ferreira, 2007). This suggests that isobutyraldehyde is a major fermentation volatile which is nearly completely (>99%) lost by evaporation. Similar considerations applied to isopropyl and isoamyl acetates and ethyl propanoate indicate that more than 70% of these aromas are lost by evaporation. Levels purged out of higher esters, such as ethyl butyrate, isobutyrate, hexanoate, octanoate and decanoate, and of higher aldehydes, such as 2 and 3-methylbutanal, were more modest. Yet, they represent significant fractions of the total formed. In the cases of ethyl hexanoate and decanoate, for instance, the fractions lost are 68 and 35% of the total amounts formed, respectively.

In any case, results reveal that the CO_2_ released during fermentation carried out large amounts of isobutyraldehyde, isopropyl acetate, ethyl propanoate and isoamyl acetate, which in fact are mostly lost in this period; and also significant amounts in absolute but not in relative terms, of isobutanol and isoamyl alcohol. Some other esters, such as ethyl butyrate, hexanoate, octanoate and decanoate were produced at much smaller levels but yet, were significantly lost by evaporation.

### 3.3 Major fermentative aroma compounds

Twenty-six major fermentation volatiles were detected at concentrations superior to detection limits in young wines (Table S2). In this case, fifteen out of the twenty-six quantified volatiles were significantly related to the yeast strain. The most different profiles of volatiles were obtained in wines fermented with IONYS, which produced maxima levels of most ethyl esters and acetates, and also of isovaleric acid, acetoin and γ-butyrolactone, and minima levels of acetic and decanoic acids. Other strains showing specific profiles of volatiles were 71B, BDX, D80, and PERSY. 71B produced maxima levels of methionol, butanol, hexanol and minima levels of most ethyl esters. BDX produced maxima levels of isobutanol and isoamyl alcohol. D80 produced maxima levels of acetic, isobutyric and decanoic acids. Finally, PERSY produced maxima levels of ethyl octanoate, octanoic acids, ethyl lactate and minima levels of isoamyl alcohol and isobutanol.

In general, differences introduced by the strains were of little to moderate magnitude. Levels of isoamyl alcohol ranged from 160 to 310 mg/L, a factor 2; those of isobutanol from 20 to 60 mg/L, a factor 3. These differences are, however, large enough to have sensory significance (De-la-Fuente-Blanco, Sáenz-Navajas, & Ferreira, 2017). Differences in the levels of acetic acid were much higher and amounted to a factor 20, from just 30 mg/L (IONYS) to 600 mg/L (RHONE). However, leaving aside IONYS, differences become more modest, ranging from 340 to 600 mg/L, less than a factor 2. Similar ranges of variation were observed for hexanoic, octanoic, decanoic and isovaleric acids, while levels of isobutyric acid ranged a factor close to 4. Levels of esters were, in general, very low as a possible consequence of their strong evaporation, and in some cases, they were not even detected. Leaving aside IONYS, their ranges of variation were not large.

### 3.4 Trace aroma compounds. Varietal or fermentative origin?

The complete data sets with the concentrations of up to thirty-four trace aroma components in recently fermented and in aged wines, in the different controls introduced in the study, and the results of the different ANOVA studies carried out on the data are compiled in the Supplementary material (Tables S2–S6).

Regarding the varietal or fermentative origin of the aroma compounds, the study of the controls including or not the PAF material extracted from the grapes and those others including or not fermentation (all compiled in Table S3), reveals that some aroma compounds cannot be unequivocally classified into fermentative or varietal. Rather, there are several intermediate categories, as the answer to the following three simple questions asked to each aroma compound, reveals;

1. Is the aroma compound present in fermented controls not containing grape PAF?
2. Is it present in unfermented controls containing grape PAF?
3. Is it at significantly larger amounts in fermented samples containing grape PAF than in the corresponding controls not containing grape PAF?

**Fig. 1:**
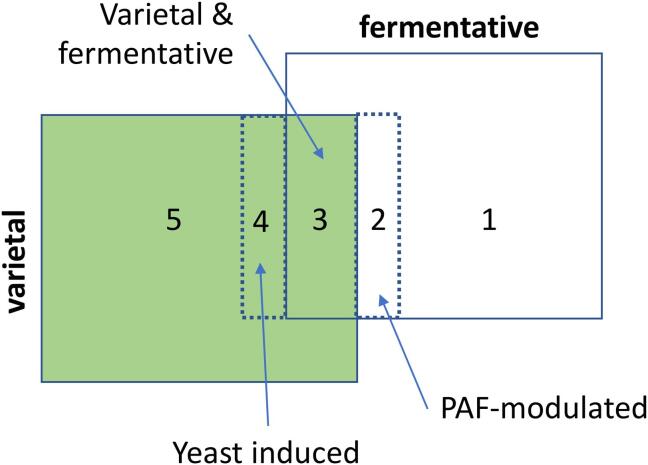
The five origin-related categories in which aroma compounds of the experiment should be classified.

A positive answer to the first question implies a unequivocal fermentative origin; the compound can be formed by yeasts from the basic list of nutrients supplied. On the contrary, a negative answer indicates that the formation of the compound requires the presence of grape components. The answers to the two following questions will indicate whether the compound is present in the grape PAF as specific precursor (positive answer to 2) and whether yeast is required for its formation (positive answer to 3). Attending to the answers, five different origin-related categories emerged, as is schematized in Fig. 1.

1) Pure fermentative compounds (answers YNN) are those which were present in fermented samples not containing PAF and whose levels were not influenced by the presence of PAF. Compounds in this category were isobutyl acetate, ethyl isovalerate, ethyl 2-methylbutyrate and γ-decalactone.
2) PAF-modulated fermentative compounds (answers YNY) are those aroma compounds formed by yeast, but whose levels are significantly influenced by the presence of PAF. Compounds in this category were β-phenylethyl acetate, ethyl isovalerate, ethyl leucate, γ-octalactone, β-citronellol, geraniol, nerol.
3) Fermentative and varietal aroma compounds (answers YYY and YYN), are those aroma compounds which can be formed by yeast from basic nutrients and which can be also found in unfermented controls containing PAF. Linalool and linalool oxide belong to this category.
4) Yeast-induced varietal aroma compounds (answers NNY), which are those aroma compounds found exclusively in fermented PAF. Ethyl dihydrocinnamate and β-ionone belong to this category.
5) Varietal aroma compounds (answers NYY), which are those compounds found exclusively in samples containing grape PAF. Most compounds belong to this category (massoia lactone, β-damascenone, TDN, vitispirane, Riesling acetal, vanillin, acetovanillone, syringaldehyde, syringol, guaiacol, 4-ethyguaiacol, 4-ethylphenol, 4-vinylguaiacol, 4-vinylphenol, eugenol, methoxyeugenol, *trans*-isoeugenol, p-propylguaiacol).

It should be noted that, directly or indirectly, levels of all compounds were influenced by the existence of fermentation, which suggests, as it will be futher seen in the next sections, that yeast is going to play a relevant role on nearly the complete wine aroma profile.

### 3.5 Yeast strain and aging: global overview

**Fig. 2:**
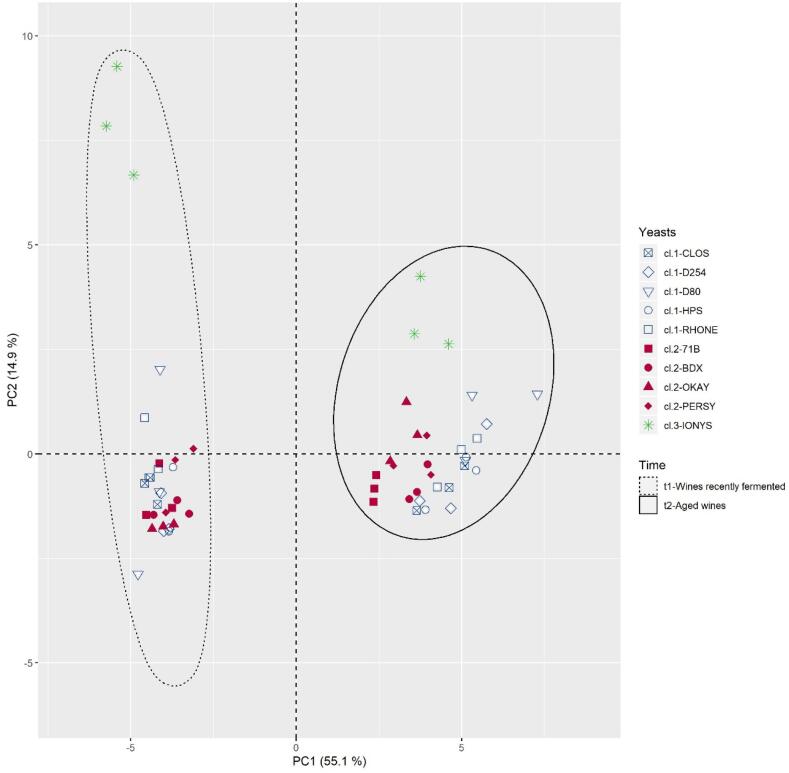
PCA carried out on concentrations of trace aroma compounds in the PAF-containing samples fermented by 10 *S. cerevisiae* strains, analysed after fermentation and after accelerated aging.

Both factors, yeast strain and aging have a strong influence on the trace aroma composition of wines, although aging is the dominant factor. Such dominance is most evident in the PCA plane given in Fig. 2, which shows the projection of the 60 samples (10 yeasts × 2 times of analysis × 3 replicates) in the two first dimensions. The first component (55.1% of the original variance) separates samples attending to age, with young samples on the left, and aged samples on the right. The variable loadings (not shown) reveals that most compounds, including norisoprenoids, esters, volatile phenols, vanillin derivatives, increase during aging and that only terpenes (except for linalool oxide), massoia lactone and phenylethyl acetate decrease during aging.

The plot reveals two other important characteristics. First, that samples fermented with IONYS are very well separated from the others before and after aging. Young samples because of its highest levels in β-phenylethyl acetate, linalool and geraniol, and aged samples because of its highest levels of isobutyl acetate, γ-octa and decalactone. The second remarkable characteristic, is that leaving aside IONYS, the yeast strain is an active grouping factor in trace aroma compounds only after aging.

### 3.6 Effects of yeast strain on wine aroma

**Fig. 3:**
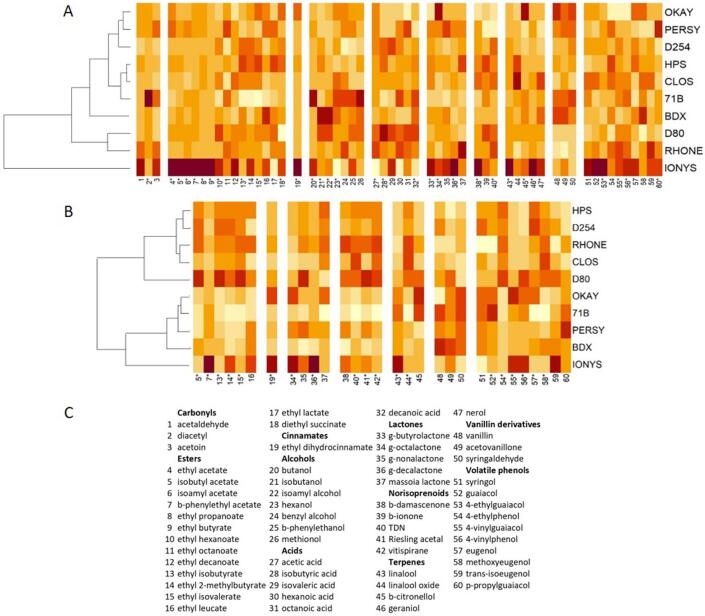
Heatmaps obtained from normalized data of the main volatiles significantly modulated by yeasts in young (A) and aged (A) wines. Compounds are detailed in the subfigure C.

In order to analyse the influence of yeast taking into consideration the dominance of the aging time, two different heatmaps were generated with aroma compounds significantly affected by yeasts. The first is given in Fig. 3A and includes data from major and trace aroma compounds measured in young wines. The second can be seen in Fig. 3B and includes only trace aroma compounds in aged wines (Fig. 3B). The results of the hierarchical clusterings are also displayed on the left part of each plot.

Fig. 3A confirms the singularity of IONYS in young wines and the apparently low diversity existent between the other strains. After aging, however, a much clear structure emerges as can be seen in Fig. 3B, where yeast strains can be classified into three different clusters. Cluster 1 is integrated by a quite homogeneous group formed by D254, HPS, RHONE and CLOS and with more dissimilarity, by D80. The second cluster is formed by PERSY, OKAY, 71B and with more dissimilarity, BDX. And finally, IONYS is the single component of the most different cluster 3. It can also be seen that yeasts in cluster 1 produced higher levels of pure fermentative compounds such as isobutyl acetate, ethyl isobutyrate, 2-methylbutyrate and isovalerate and released higher levels of norisoprenoids (TDN, vitispirane and Riesling acetal). Cluster 2 released in general smaller levels of volatiles, except for some volatile phenols, such as vanillin and guaiacol.

### 3.7 Modulation of varietal aroma

**Fig. 4:**
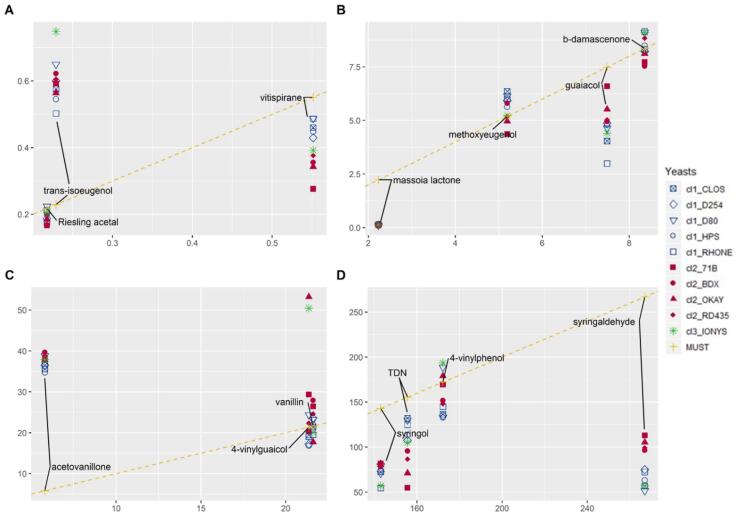
Concentrations of varietal aroma compounds in PAF-containing aged fermented samples according to their levels in the unfermented control.

The effect of yeast strain on those genuine varietal aroma compounds naturally present in unfermented controls containing grape PAF can be assessed with the help of the plots given in Fig. 4. The plots compare levels of aroma compounds found in fermented aged samples with those obtained in the unfermented aged controls. These representations facilitate the identification of the general role of fermentation on the fate of these varietal aroma compounds and also of the specific role played by the strain of yeast. Compounds can be classified into three different categories depending on the effect of fermentation.

1st category.- Positive effect of fermentation. This category includes five aroma compounds whose levels in fermented samples were much above those found in the unfermented controls, suggesting that some of the specific precursors of these aroma compounds could be formed by the action of yeasts. Compounds in this category are acetovanillone, β-ionone (this one is not shown in Fig. 4, since it was only found in young samples), ethyl dihydrocinnamate, p-propylguaiacol, 4-ethylphenol, *trans*-isoeugenol (these four are not represented in Fig. 4 since they were not detected in the unfermented control) and eugenol. Only in the case of eugenol the effect of yeast was significant. Levels of acetovanillone in aged fermented samples are 10 times higher than those found in the unfermented control, however, no difference was observed between the yeasts.

2nd category.- No effect of fermentation. Compounds in this category have in common that, in average, levels of fermented samples are not dissimilar to those of the unfermented controls. There are however strong differences attending to the differential effect introduced by yeast. In the cases of Riesling acetal in Fig. 4A, and of methoxyeugenol and β-damascenone in Fig. 4B, there is no effect of yeast, which suggests, that at least for Tempranillo, the levels of these varietal aroma compounds cannot be modulated by yeast. The case of β-damascenone, which is an aroma enhancer (Pineau, Barbe, Van Leeuwen, & Dubourdieu, 2007) and modules the ripeness-character of fruity perception (San-Juan, Ferreira, Cacho, & Escudero, 2011), deserves special mention. Levels of this odorant in recently fermented samples were above those measured in the corresponding unfermented controls (Supplementary material S2 and S5) and were significantly influenced by the strain of yeast. Samples fermented with IONYS showed levels up to 4 times higher than those found in those made with OKAY. This suggests that yeasts cannot change the long-term level of this aroma compound but can accelerate its formation. The second subcategory includes vanillin (4C), methoxyeugenol (4B), and 4-vinylphenol (4D), for which the strain of yeast exerts a moderate and significant influence, so that differences of around a 50% between the minimum and the maximum are observed. 4-Vinylguaiacol can be also classified within this subcategory but with two strains, IONYS and OKAY, showing a clear outlier overproductive character.

3rd category.- Negative effect of fermentation. This category includes aroma compounds whose levels in fermented samples were below those found in the unfermented controls. There are strong differences between compounds attending to the effect played by the strain of yeast. Massoia lactone (Fig. 4B) is a particular case whose levels drop to nearly zero in all fermented samples with no difference between strains. Massoia lactone is an important marker of over-ripeness and contributor to prune aroma, whose levels are known to decrease during fermentation (Pons, Allamy, Lavigne, Dubourdieu, & Darriet, 2017). Our results reveal that such decrease intensifies during aging, which suggests that fermentation reduces also the precursors. It would be of interest to see whether such reduction is equally effective in grapes containing higher levels of precursors of this molecule. Compounds in the category for which the strain of yeast introduced significant differences were, from less to more intense the effect of strain: syringol, vitispirane, syringaldehyde, guaiacol and TDN. It is apparent that the specific precursors of these compounds are metabolized differently by the different strains. This can have strong technological relevance since guaiacol and TDN can take part in relevant odour faults, and suggests that selected strains are a potentially effective remedial tool. Guaiacol is an aroma compound contributing to the characteristic toasty-woody notes of Tempranillo, but it can be a serious off-odour developed with time in wines made with grapes exposed to smoke (Ristic, Van Der Hulst, Capone, & Wilkinson, 2017). Results in Fig. 4 reveal that yeasts within the cluster 1 seem to metabolize the precursor at higher levels. In the case of RHONE, levels of guaiacol were reduced by almost a factor 3 comparing with the unfermented control. Powerful reductions linked to specific yeast strains can be also observed for TDN, known responsible for kerosene notes developed in aged Riesling wines. With a 2 μg/L detection threshold (Sacks et al., 2012), this compound will surely also contribute to unpleasant notes in aged red wines. The figure also reveals that yeasts in cluster 2, notably 71B can TDN reduce levels by a factor 3 with respect to the control or D80. A similar yeast-induced and vitispirane-independent decrease of TDN levels has been recently observed for non-*Saccharomyces* yeast (Oliveira & Ferreira, 2019) but to the best of our knowledge, it has not been observed for *Saccharomyces* strains. This ability can have a notable sensory importance, since levels of TDN are expected to increase due to climate change (Winterhalter & Gök, 2013).

Many varietal aroma compounds in Fig. 4 derive from ferulic acid and its glycosides (vanillin, acetovanillone, isoeugenol, eugenol and 4-vinylguaiacol). The higher levels of these aroma molecules measured in aged fermented samples suggest that yeast transforms ferulic acid glycosides into the corresponding aroma glycosides and that those transformations are strain specific. Aged wines made with BDX have maxima contents of vanillin and acetovanillone, those made with IONYS have maxima contents of eugenol while those made with OKAY have minima levels of vanillin and maxima levels of 4-vinylguaiacol. Some of those specificities seem to be shared by strains in the same cluster. Yeasts in cluster 2 show higher levels of vanillin, acetovanillone and 4-vinylguaiacol than those in cluster 1. 4-Vinylguaiacol and 4-vinylphenol deserve a specific comment, since their levels are extremely dependent of the yeast strain in unaged samples, with factors around 10 between the minimum and maximum concentrations. Because of their reactivity, differences between maxima and minima shrink to factors 3 (case of 4-vinylguaiacol) and 1.7 (case of 4-vinylphenol). In both cases, maxima levels were observed for IONYS, and minima levels for BDX and HPS.

### 3.8 Modulation of other relevant aroma molecules

Linalool and geraniol are the two most important terpenols of wine. In the present case, these two molecules were hardly detected in the unfermented controls, suggesting that the grape material did not have much precursors. The two compounds were found in the controls not containing grape extract, so that yeasts were able to form weak, or moderate in the case of IONYS, amounts of these molecules. Fermented unaged samples contained both molecules at the expected concentration ranges of Tempranillo (<6 μg/L) except samples fermented by IONYS, whose levels were above 20 μg/L, which suggests that this quite unique yeast strain will produce young wines with markedly different characters. During aging, levels of these compounds and the other terpenes decreased (<1 μg/L), while those of linalool oxide, its oxidation product, increased.

Ethyl leucate or ethyl 2-hydroxy-4-methylvalerate, is a remarkable aroma compound identified in aged wines (Campo, Cacho, & Ferreira, 2006) and suggested to be key in the specific blackberry aroma of Bordeaux red wines (Falcao, Lytra, Darriet, & Barbe, 2012). Results from this paper have shown that maxima levels are found in aged PAF-containing fermented samples and that the yeast strain exerts a significant influence, with levels found in IONYS, BDX, D80, PERSY and RHONE twice those found in 71B or OKAY (Table S5).

Ethyl esters of branched acids are the most important fruity esters in aged red wines, contributing concertedly to fruity aroma (De-la-Fuente-Blanco, Sáenz-Navajas, Valentin, & Ferreira, 2020) and are slowly formed by esterification of the corresponding acids synthesized during fermentation through Ehrlich pathway (Swiegers, Bartowsky, Henschke, & Pretorius, 2005). The influence of yeast becomes most obvious after aging. Minima levels are found in 71B, PERSY (cluster 2), and maxima in D80 and D254 (cluster 1), with differences as large as factors between 4 and 6.5.

Isoamyl and phenylethyl acetates are relevant in the aroma of young wines, since their levels quickly fade by hydrolysis of the esters. The influence of yeasts in their levels is overwhelming. In the case of β-phenylethyl acetate, levels in young wines (Table 5, Supplementary material) range from 0.1 mg/L (cluster 1) to 1.5 mg/L in cluster 3, with levels in samples from cluster 2 between 0,18 and 0.29 mg/L. Similar results were already observed for the isoamyl acetate and isopropyl acetates evaporated during fermentation (Table 2).

## 4 Conclusions

Volatiles lost by evaporation during fermentation are mostly fermentative compounds and not grape-related aroma compounds. Quantitatively, vapours are majorly composed of 2-methylpropanal, isopropyl acetate, isoamyl acetate, ethyl propanoate, isobutanol and isoamyl alcohol. While the fraction of alcohols lost is very low, that of the aldehydes and esters can be well above 90% of the total volatile produced.

The strong impact exerted by the strain of yeast on wine aroma composition becomes in many cases only evident after aging, since levels of ethyl esters of branched acids, of most grape-related aroma compounds and of many minor yeasts-derived aroma compounds mostly increase during aging. The 10 strains can be classified into three clusters showing marked differences in fermentative and varietal aroma profiles.

The boundaries between fermentative and varietal aroma compounds are in many cases blurred. First, the study has confirmed a fermentative origin for linalool and geraniol, found at high levels in samples fermented by one of the strains. Second, the presence of polyphenolic and aromatic fractions from grape exerts a strong influence on yeast metabolism and, third, the strains of yeast not only hydrolyze glycosidic precursors, but metabolize quite differently the precursors of relevant aroma compounds, such as phenolic acids and norisoprenoids. These characteristics have interesting practical consequences on the potential of yeasts to control the wine aroma profile and, most remarkably, some wine aging attributes. Results have shown that the rates of accumulation of β-damascenone are strain-related, and that some strains may be specifically used to mitigate relevant aging-related off-odours, such as those related to guaiacol, massoia lactone or TDN.

## CRediT authorship contribution statement

**Marie Denat:** Investigation, Formal analysis, Data curation, Writing - original draft, Visualization. **Dolores Pérez:** Investigation, Writing - review & editing. **José María Heras:** Conceptualization, Resources, Writing - review & editing. **Amparo Querol:** Supervision, Methodology, Writing - review & editing. **Vicente Ferreira:** Supervision, Validation, Methodology, Writing - review & editing, Visualization.

## Declaration of Competing Interest

The authors declare that they have no known competing financial interests or personal relationships that could have appeared to influence the work reported in this paper.
