# Supplementary material for: The effects of Saccharomyces cerevisiae strains carrying alcoholic fermentation on the fermentative and varietal aroma profiles of young and aged Tempranillo wines
Source: Food Chem X. 2021 Feb 9;9:100116. doi: 10.1016/j.fochx.2021.100116 (PMC7902897; doi:10.1016/j.fochx.2021.100116)
Supplement: Supplementary data 1 [file mmc1.docx]

Figure S1: Experimental procedure


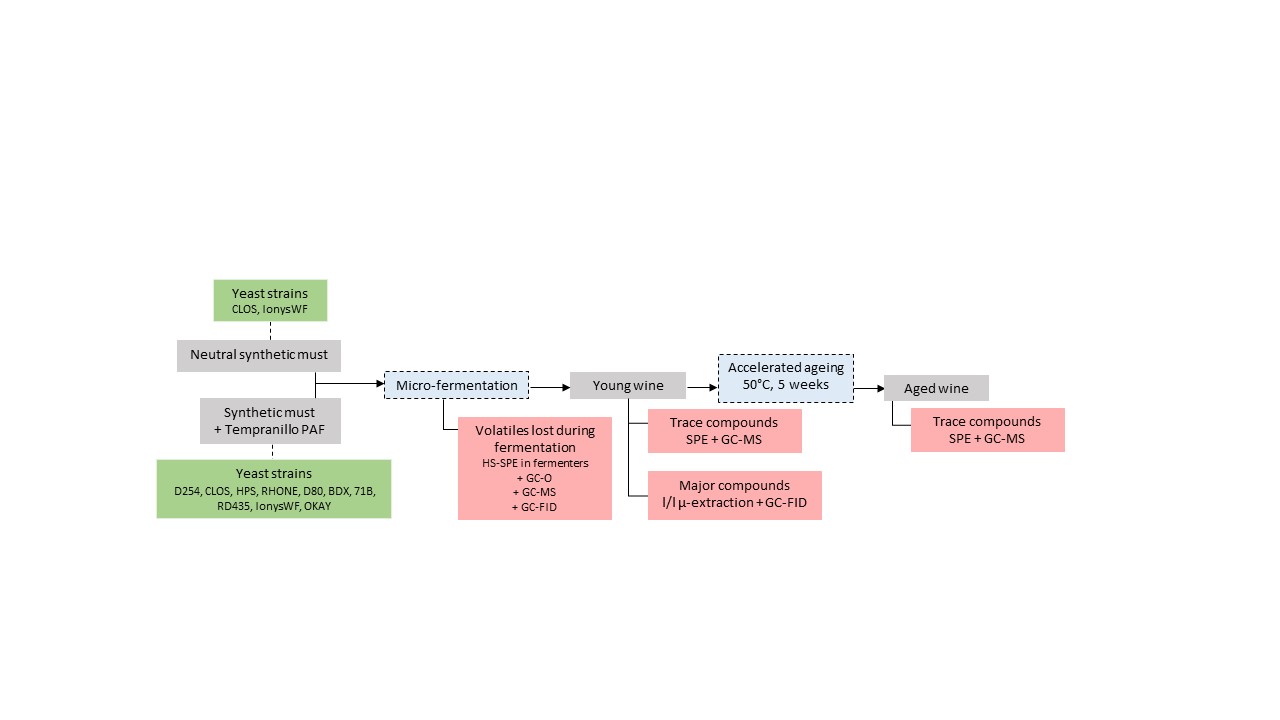


| Table S1: List of the compounds quantified in this study. In the case of a GC-MS quantification m/z ratio are provided, the first one was used for quantification. | | | |
| --- | --- | --- | --- |
| Compounds | | CAS | m/z |
| **Additional fermentative compounds (GC-MS)** | | | |
| isobutyraldehyde | | 78-84-2 | 57, 55, 71 |
| 2-methylbutanal | | 96-17-3 | 57, 58 |
| 3-methylbutanal | | 590-86-3 | 58, 57 |
| propyl acetate | | 109-60-4 | 61, 73 |
| isopropyl acetate | | 108-21-4 | 61, 59 |
| isobutyl acetate | | 110-19-0 | 73, 86 |
| ethyl isobutyrate | | 97-62-1 | 71, 116 |
| 2-methylbutyric acid | | 116-53-0 | 74, 57 |
| 3-methylbutyric acid | | 503-74-2 | 60, 87 |
| **Major volatile compounds (GC-FID)** |  | | |
| acetaldehyde | | 75-07-0 |  |
| diacetyl | | 431-03-8 |  |
| acetoin | | 513-86-0 |  |
| ethyl acetate | | 141-78-6 |  |
| isoamyl acetate | | 123-92-2 |  |
| hexyl acetate | | 142-92-7 |  |
| ethyl propanoate | | 105-37-3 |  |
| ethyl butyrate | | 105-54-4 |  |
| ethyl hexanoate | | 123-66-0 |  |
| ethyl octanoate | | 106-32-1 |  |
| ethyl decanoate | | 110-38-3 |  |
| ethyl lactate | | 97-64-3 |  |
| diethyl succinate | | 123-25-1 |  |
| g-butyrolactone | | 96-48-0 |  |
| acetic acid | | 64-19-7 |  |
| butyric acid | | 107-92-6 |  |
| isobutyric acid | | 79-31-2 |  |
| isovaleric acid | | 503-74-2 |  |
| hexanoic acid | | 142-62-1 |  |
| octanoic acid | | 124-07-2 |  |
| decanoic acid | | 334-48-5 |  |
| 1-butanol | | 71-36-3 |  |
| isobutanol | | 78-83-1 |  |
| isoamyl alcohol | | 123-51-3 |  |
| 1-hexanol | | 111-27-3 |  |
| cis-3-hexenol | | 928-96-1 |  |
| benzyl alcohol | | 100-51-6 |  |
| 2-phenylethanol | | 60-12-8 |  |
| methionol | | 505-10-2 |  |
| **Trace volatile compounds (GC-MS)** |  | | |
| isobutyl acetate | | 110-19-0 | 56, 101, 73 |
| b-phenylethyl acetate | | 103-45-7 | 89, 91 |
| ethyl isobutyrate | | 97-62-1 | 71, 116 |
| ethyl 2-methylbutyrate | | 7452-79-1 | 57, 102, 115 |
| ethyl isovalerate | | 108-64-5 | 88, 115, 70 |
| ethyl 4-methylvalerate | | 25415-67-2 | 88, 101, 115 |
| ethyl cyclohexanoate | | 3289-28-9 | 83, 101, 156 |
| ethyl D/L-leucate | | 10348-47-7 | 69, 117, 87 |
| trans-ethyl cinnamate | | 103-36-6 | 131, 103, 176 |
| ethyl dihydrocinnamate | | 2021-28-5 | 178, 149, 133 |
| R/S-g-octalactone | | 104-50-7 | 85, 114, 100 |
| g-nonalactone | | 104-61-0 | 85, 128, 100 |
| g-decalactone | | 706-14-9 | 85, 128, 100 |
| massoia lactone | | 54814-64-1 | 97, 139, 68 |
| cis-whiskylactone | | 55013-32-6 | 99, 100, 114 |
| trans-whiskylactone | | 39638-67-0 | 99, 114, 128 |
| a-ionone | | 127-41-3 | 121, 93, 192 |
| b-ionone | | 201-224-3 | 177, 135, 192 |
| (+)-cis/trans-rose oxide | | 16409-43-1 | 139, 140, 154 |
| b-damascenone | | 23726-93-4 | 190, 175, 69 |
| TDN | | 30364-38-6 | 157, 142, 172 |
| Riesling acetal | | 129601-94-1 | 138, 125, 133 |
| vitispirane | | 65416-59-3 | 93, 192, 136, 121 |
| 1,8-cineole | | 470-82-6 | 108, 139, 154 |
| R-limonene | | 5989-27-5 | 107, 136, 121 |
| linalool | | 78-70-6 | 71, 93, 121 |
| cis/trans-linalool oxide | | 60047-17-8 | 94, 59, 111 |
| b-citronellol | | 106-22-9 | 69, 81, 123 |
| geraniol | | 106-24-1 | 69, 139, 123 |
| nerol | | 106-25-2 | 93, 121, 68 |
| a-terpineol | | 98-55-5 | 93, 121, 136 |
| vanillin | | 121-33-5 | 152, 151, 123 |
| acetovanillone | | 498-02-2 | 166, 151, 123 |
| syringaldehyde | | 134-96-3 | 182, 181, 167 |
| syringol | | 91-10-1 | 154, 139 |
| guaiacol | | 90-05-1 | 109, 125, 124 |
| 4-ethylguaiacol | | 2785-89-9 | 137, 122, 152 |
| 4-ethylphenol | | 123-07-9 | 107, 122 |
| 4-vinylguaiacol | | 7786-61-0 | 150, 135 |
| 4-vinylphenol | | 2628-17-3 | 120, 91 |
| m-cresol | | 108-39-4 | 108, 90, 79 |
| o-cresol | | 95-48-7 | 108, 107, 79 |
| eugenol | | 97-53-0 | 164, 149 |
| methoxyeugenol | | 6627-88-9 | 194, 179, 119 |
| trans-isoeugenol | | 97-54-1 | 164, 131, 149 |
| p-propylguaiacol | | 2785-87-7 | 137, 122, 166 |

| Table S2.- Concentrations (μg/L) of major and trace aroma compounds found in the recently fermented wines by 10 *S. cerevisiae* yeasts with PAF (mean ± standard deviation). Letters indicates Tukey test results. | | | | | | | | | | |
| --- | --- | --- | --- | --- | --- | --- | --- | --- | --- | --- |
|  | CLOS | IONYS | 71B | BDX | D254 | D80 | HPS | OKAY | PERSY | RHONE |
| **Carbonyls** |  |  |  |  |  |  |  |  |  |  |
| acetaldehyde | 8008 ± 7000 | 17910 ± 10000 | 9170 ± 900 | 8469 ± 6000 | 6646 ± 3000 | 8729 ± 4000 | 10267 ± 8000 | 9657 ± 4000 | 10636 ± 2000 | 13072 ± 4000 |
| diacetyl ^Y^ | 39 ± 30 bc | 69 ± 10 bc | 816 ± 70 a | 101 ± 10 bc | 34 ± 30 bc | 111 ± 30 b | 62 ± 20 bc | 82 ± 30 bc | 103 ± 10 b | 85 ± 20 bc |
| acetoin | 308 ± 200 | 790 ± 500 | 564 ± 60 | 300 ± 200 | 252 ± 100 | 283 ± 100 | 372 ± 300 | 385 ± 200 | 633 ± 100 | 610 ± 200 |
| **Esters** |  |  |  |  |  |  |  |  |  |  |
| ethyl acetate ^Y^ | 20985 ± 800 de | 50338 ± 3000 a | 18345 ± 2000 e | 23796 ± 600 cde | 23051 ± 3000 cde | 24684 ± 2000 cd | 24654 ± 3000 cd | 28687 ± 2000 bc | 32911 ± 3000 b | 22829 ± 1000 cde |
| isobutyl acetate ^Y^ | 8 ± 0.4 b | 76 ± 20 a | 11 ± 1 b | 22 ± 1 b | 14 ± 3 b | 15 ± 10 b | 19 ± 4 b | 10.7 ± 0.3 b | 18 ± 1 b | 14 ± 1 b |
| isoamyl acetate ^Y^ | 43 ± 10 bc | 540 ± 100 a | 115 ± 20 bc | 99 ± 20 bc | 70 ± 30 bc | 121 ± 100 bc | 98 ± 50 bc | 102 ± 30 bc | 210 ± 40 b | 115 ± 20 bc |
| b-phenylethyl acetate ^Y^ | 81 ± 20 b | 1509 ± 30 a | 256 ± 80 b | 204 ± 20 b | 83 ± 30 b | 200 ± 200 b | 117 ± 50 b | 182 ± 30 b | 283 ± 30 b | 140 ± 30 b |
| ethyl propanoate ^Y^ | 32 ± 4 b | 263 ± 100 a | n.d. b | 17 ± 10 b | 26 ± 20 b | 27 ± 5 b | 32 ± 10 b | 34 ± 10 b | 59 ± 4 b | 28 ± 2 b |
| ethyl butyrate ^Y^ | n.d. b | 6 ± 1 a | n.d. b | n.d. b | n.d. b | n.d. b | n.d. b | n.d. b | n.d. b | n.d. b |
| ethyl hexanoate ^Y^ | 51 ± 6 abc | 63 ± 10 a | 37 ± 3 c | 47 ± 10 abc | 49 ± 4 abc | 59 ± 9 ab | 44 ± 6 abc | 40 ± 2 bc | 43 ± 1 abc | 43 ± 6 abc |
| ethyl octanoate | n.d. | 12 ± 20 | 17 ± 4 | 9 ± 10 | n.d. | 11 ± 20 | 11 ± 20 | 9 ± 8 | 18 ± 2 | 17 ± 10 |
| ethyl decanoate | 192 ± 70 | 247 ± 100 | 132 ± 50 | 107 ± 40 | 151 ± 90 | 178 ± 20 | 138 ± 50 | 86 ± 80 | 148 ± 70 | 172 ± 100 |
| ethyl isobutyrate ^Y^ | 6.9 ± 0.2 ab | 5 ± 2 abc | 2.4 ± 0.7 c | 7.1 ± 0.9 ab | 8 ± 0.6 ab | 7 ± 2 ab | 9 ± 1 a | 5 ± 0.5 bc | 5 ± 1 bc | 6 ± 2 ab |
| ethyl 2-methylbutyrate ^Y^ | 0.56 ± 0.04 abc | 0.8 ± 0.2 a | 0.21 ± 0.07 c | 0.34 ± 0.06 bc | 0.7 ± 0.2 ab | 0.5 ± 0.1 abc | 0.6 ± 0.2 ab | 0.5 ± 0.1 abc | 0.5 ± 0.2 abc | 0.5 ± 0.1 abc |
| ethyl isovalerate ^Y^ | 0.83 ± 0.08 ab | 0.7 ± 0.2 ab | 0.28 ± 0.04 c | 0.7 ± 0.1 ab | 0.83 ± 0.09 ab | 0.8 ± 0.3 ab | 1 ± 0.2 a | 0.89 ± 0.09 ab | 0.5 ± 0.08 bc | 0.75 ± 0.06 ab |
| ethyl leucate | 10 ± 4 | 28 ± 8 | 6.7 ± 0.8 | 31 ± 10 | 9 ± 6 | 23 ± 30 | 17 ± 9 | 21 ± 3 | 23 ± 4 | 10 ± 2 |
| ethyl dihydrocinnamate ^Y^ | 0.021 ± 0.002 c | 0.058 ± 0.008 a | 0.018 ± 0.004 c | 0.023 ± 0.005 bc | 0.021 ± 0.001 c | 0.021 ± 0.007 c | 0.026 ± 0.001 bc | 0.036 ± 0.011 b | 0.023 ± 0.005 bc | 0.022 ± 0.003 bc |
| ethyl lactate | 378 ± 50 | 278 ± 80 | 360 ± 90 | 478 ± 50 | 441 ± 200 | 516 ± 300 | 585 ± 300 | 355 ± 40 | 538 ± 50 | 494 ± 70 |
| diethyl succinate ^Y^ | 3774 ± 3000 ab | 6938 ± 1000 a | 3660 ± 400 ab | 4566 ± 800 ab | 5086 ± 1000 ab | 665 ± 60 b | 5799 ± 3000 a | 5781 ± 3000 a | 3848 ± 100 ab | 5011 ± 500 ab |
| **Alcohols** |  |  |  |  |  |  |  |  |  |  |
| 1-butanol ^Y^ | 201 ± 30 b | 967 ± 400 a | 1135 ± 300 a | 185 ± 20 b | 165 ± 40 b | 160 ± 40 b | 179 ± 90 b | 234 ± 20 b | 294 ± 40 b | 186 ± 20 b |
| isobutanol ^Y^ | 28613 ± 3000 c | 33352 ± 3000 bc | 22289 ± 1000 c | 61885 ± 9000 a | 36045 ± 6000 bc | 50817 ± 10000 ab | 37738 ± 8000 bc | 20366 ± 2000 c | 20029 ± 4000 c | 32779 ± 3000 c |
| isoamyl alcohol ^Y^ | 194852 ± 20000 ab | 217380 ± 40000 ab | 205485 ± 20000 ab | 306583 ± 30000 a | 173993 ± 40000 ab | 266544 ± 1e+05 ab | 191365 ± 70000 ab | 187005 ± 20000 ab | 161094 ± 6000 b | 189807 ± 20000 ab |
| 1-hexanol ^Y^ | 25 ± 6 a | 17 ± 1 b | 25.5 ± 0.5 a | 24 ± 1 ab | 23 ± 3 ab | 22 ± 3 ab | 20 ± 3 ab | 23 ± 1 ab | 21 ± 1 ab | 21.9 ± 0.9 ab |
| benzyl alcohol | 109 ± 40 | 78 ± 60 | 123 ± 70 | 101 ± 20 | 61 ± 10 | 101 ± 20 | 93 ± 30 | 105 ± 40 | 104 ± 4 | 126 ± 40 |
| 2-phenylethanol | 21393 ± 10000 | 43029 ± 20000 | 45053 ± 20000 | 34184 ± 10000 | 14929 ± 8000 | 40498 ± 20000 | 19262 ± 7000 | 29549 ± 6000 | 34806 ± 20000 | 26471 ± 3000 |
| methionol | 4880 ± 2000 | 5370 ± 3000 | 11721 ± 4000 | 8741 ± 3000 | 4154 ± 1000 | 8873 ± 3000 | 6413 ± 4000 | 6125 ± 2000 | 5066 ± 2000 | 6230 ± 2000 |
| **Acids** |  |  |  |  |  |  |  |  |  |  |
| acetic acid ^Y^ | 426171 ± 40000 a | 35446 ± 5000 b | 407025 ± 70000 a | 372495 ± 70000 a | 494160 ± 70000 a | 548806 ± 2e+05 a | 427292 ± 60000 a | 462674 ± 2e+05 a | 453883 ± 2e+05 a | 611486 ± 1e+05 a |
| isobutyric acid ^Y^ | 3864 ± 800 bcd | 2931 ± 200 bcde | 1878 ± 200 de | 3895 ± 800 bcd | 5455 ± 400 ab | 7190 ± 3000 a | 4921 ± 200 abc | 2150 ± 600 cde | 2249 ± 500 cde | 4310 ± 1000 bcd |
| isovaleric acid | 141 ± 100 | 242 ± 60 | 89 ± 20 | 129 ± 70 | 223 ± 80 | 214 ± 2 | 116 ± 40 | 85 ± 70 | 88 ± 20 | 149 ± 50 |
| hexanoic acid | 290 ± 100 | 182 ± 90 | 355 ± 200 | 244 ± 60 | 317 ± 300 | 329 ± 30 | 284 ± 100 | 231 ± 200 | 322 ± 80 | 367 ± 80 |
| octanoic acid | 111 ± 4 | 141 ± 20 | 129 ± 20 | 102 ± 10 | 112 ± 20 | 154 ± 20 | 128 ± 20 | 67 ± 50 | 159 ± 40 | 146 ± 20 |
| decanoic acid ^Y^ | 194 ± 30 ab | n.d. b | 420 ± 80 a | 358 ± 100 a | 216 ± 60 ab | 421 ± 300 a | 312 ± 100 ab | 168 ± 100 ab | n.d. b | 261 ± 60 ab |
| **Lactones** |  |  |  |  |  |  |  |  |  |  |
| g-butyrolactone ^Y^ | 533 ± 100 b | 928 ± 200 a | 200 ± 50 cde | 387 ± 50 bcd | 455 ± 100 bc | 326 ± 70 bcde | 495 ± 200 bc | 102 ± 9 de | 481 ± 30 bc | 454 ± 40 bc |
| g-octalactone ^Y^ | 0.29 ± 0.09 b | 2 ± 1 ab | 0.4 ± 0.1 b | 0.79 ± 0.08 ab | 0.7 ± 0.2 b | 1 ± 1 ab | 0.9 ± 0.5 ab | 2.3 ± 0.2 a | 1.3 ± 0.1 ab | 0.33 ± 0.09 b |
| g-nonalactone | 2 ± 0.07 | 2.3 ± 0.4 | 1.9 ± 0.2 | 2.1 ± 0.2 | 2 ± 0.1 | 2 ± 0.6 | 2.1 ± 0.3 | 2 ± 0.2 | 2.2 ± 0.3 | 2.1 ± 0.1 |
| g-decalactone ^Y^ | 0.9 ± 0.1 bcd | 1.9 ± 0.1 a | 0.88 ± 0.09 bcd | 0.65 ± 0.08 d | 0.82 ± 0.03 cd | 0.77 ± 0.01 d | 0.9 ± 0.1 bcd | 0.85 ± 0.08 bcd | 1.1 ± 0.2 b | 1.1 ± 0.1 bc |
| massoia lactone | 0.57 ± 0.05 | 0.6 ± 0.2 | 0.5 ± 0.1 | 0.6 ± 0.2 | 0.58 ± 0.06 | 0.52 ± 0.04 | 0.68 ± 0.06 | 0.6 ± 0.1 | 0.6 ± 0.2 | 0.8 ± 0.1 |
| **Norisoprenoids** |  |  |  |  |  |  |  |  |  |  |
| b-damascenone ^Y^ | 2.8 ± 0.7 b | 4.3 ± 0.7 a | 1.5 ± 0.2 cd | 1.82 ± 0.09 bcd | 2 ± 0.2 bcd | 2 ± 0.6 bcd | 2.4 ± 0.5 bc | 1.1 ± 0.3 d | 2.1 ± 0.3 bcd | 2.1 ± 0.3 bcd |
| b-ionone | 0.208 ± 0.046 | 0.27 ± 0.22 | 0.21 ± 0.16 | 0.151 ± 0.096 | 0.14 ± 0.004 | 0.28 ± 0.11 | 0.30 ± 0.19 | 0.120 ± 0.047 | 0.054 ± 0.023 | 0.163 ± 0.009 |
| TDN ^Y^ | 2.6 ± 0.2 abc | 2 ± 1 abc | 2 ± 1 abc | 1.6 ± 0.9 abc | 2.9 ± 0.4 ab | 2.6 ± 0.2 abc | 2.6 ± 0.4 abc | 1.2 ± 0.6 bc | 0.9 ± 0.5 c | 3.2 ± 0.1 a |
| **Terpenes** |  |  |  |  |  |  |  |  |  |  |
| linalool ^Y^ | 4 ± 0.3 b | 22 ± 6 a | 1.5 ± 0.2 b | 4 ± 0.6 b | 3.61 ± 0.03 b | 5 ± 1 b | 4 ± 1 b | 1.75 ± 0.01 b | 2.29 ± 0.05 b | 3.9 ± 0.3 b |
| linalool oxide | 0.5 ± 0.4 | 0.39 ± 0.05 | 0.32 ± 0.06 | 0.29 ± 0.05 | 0.3 ± 0.03 | 0.32 ± 0.04 | 0.46 ± 0.09 | 0.26 ± 0.05 | 0.27 ± 0.08 | 0.22 ± 0.06 |
| b-citronellol ^Y^ | 2.3 ± 0.3 bc | 4 ± 1 ab | 3.4 ± 0.2 b | 3.12 ± 0.08 bc | 2.2 ± 0.1 bc | 3 ± 0.9 bc | 2.4 ± 0.3 bc | 5 ± 0.6 a | 1.6 ± 0.2 c | 2.3 ± 0.2 bc |
| geraniol ^Y^ | 6.2 ± 0.5 b | 21 ± 2 a | 3.9 ± 0.4 c | 3.3 ± 0.4 c | 2.9 ± 0.5 c | 4.1 ± 0.9 c | 2.5 ± 0.1 c | 3.7 ± 0.4 c | 3.99 ± 0.07 c | 4 ± 0.5 c |
| nerol ^Y^ | 0.9 ± 0.1 ab | 0.97 ± 0.08 a | 0.77 ± 0.02 ab | 0.85 ± 0.06 ab | 0.72 ± 0.01 ab | 0.8 ± 0.2 ab | 0.8 ± 0.1 ab | 0.75 ± 0.03 ab | 0.67 ± 0.02 b | 0.74 ± 0.06 ab |
| **Vanillin derivatives** |  |  |  |  |  |  |  |  |  |  |
| vanillin | 5 ± 2 | 6 ± 3 | 10 ± 6 | 10 ± 3 | 6 ± 2 | 5 ± 1 | 7 ± 1 | 11 ± 5 | 9 ± 3 | 4 ± 1 |
| acetovanillone | 36 ± 4 | 37 ± 3 | 38 ± 1 | 40 ± 3 | 35 ± 2 | 35 ± 7 | 35 ± 2 | 39 ± 2 | 39 ± 2 | 33.1 ± 0.1 |
| syringaldehyde | 17 ± 20 | 11 ± 9 | 64 ± 30 | 39 ± 20 | 42 ± 7 | 10 ± 2 | 36 ± 20 | 66 ± 40 | 54 ± 20 | 11 ± 10 |
| **Volatile phenols** |  |  |  |  |  |  |  |  |  |  |
| syringol | 4 ± 1 | 4 ± 2 | 3.6 ± 0.5 | 3.3 ± 0.2 | 3.4 ± 0.5 | 3.1 ± 0.7 | 3.11 ± 0.09 | 3.46 ± 0.08 | 3.3 ± 0.1 | 3.4 ± 0.9 |
| guaiacol | 0.8 ± 0.4 | 1.1 ± 0.9 | 0.6 ± 0.1 | 0.58 ± 0.01 | 0.7 ± 0.2 | 0.6 ± 0.2 | 0.62 ± 0.05 | 0.65 ± 0.01 | 0.53 ± 0.03 | 0.7 ± 0.3 |
| 4-ethylguaiacol ^Y^ | 0.108 ± 0.009 b | 0.37 ± 0.08 a | 0.076 ± 0.002 b | 0.027 ± 0.002 b | 0.08 ± 0.02 b | 0.12 ± 0.04 b | 0.06 ± 0.04 b | 0.06 ± 0.01 b | 0.1 ± 0.02 b | 0.07 ± 0.06 b |
| 4-ethylphenol | 0.17 ± 0.04 | 0.16 ± 0.01 | 0.154 ± 0.003 | 0.163 ± 0.006 | 0.152 ± 0.008 | 0.14 ± 0.04 | 0.178 ± 0.008 | 0.134 ± 0.005 | 0.17 ± 0.01 | 0.17 ± 0.01 |
| 4-vinylguaicol ^Y^ | 28 ± 7 ab | 40 ± 30 a | 27 ± 8 ab | 14 ± 6 ab | 28 ± 9 ab | 29 ± 9 ab | 22 ± 3 ab | 3.7 ± 0.8 b | 18 ± 4 ab | 39 ± 4 a |
| 4-vinylphenol ^Y^ | 163 ± 50 ab | 280 ± 90 a | 199 ± 9 ab | 118 ± 20 bc | 111 ± 20 bc | 177 ± 90 ab | 96 ± 10 bc | 25 ± 0.8 c | 106 ± 20 bc | 220 ± 7 ab |
| eugenol | 0.42 ± 0.05 | 0.53 ± 0.07 | 0.41 ± 0.06 | 0.49 ± 0.02 | 0.48 ± 0.02 | 0.4 ± 0.1 | 0.51 ± 0.04 | 0.51 ± 0.05 | 0.43 ± 0.04 | 0.43 ± 0.04 |
| methoxyeugenol | 1.2 ± 0.3 | 1.1 ± 0.3 | 1.1 ± 0.5 | 1.3 ± 0.5 | 1 ± 0.3 | 0.9 ± 0.4 | 1.1 ± 0.5 | 1.2 ± 0.4 | 1 ± 0.4 | 1.1 ± 0.4 |
| trans-isoeugenol | 0.7 ± 0.1 | 0.8 ± 0.2 | 0.6 ± 0.1 | 0.65 ± 0.06 | 0.7 ± 0.2 | 0.6 ± 0.2 | 0.7 ± 0.1 | 0.64 ± 0.03 | 0.59 ± 0.07 | 0.8 ± 0.1 |
| p-propylguaiacol ^Y^ | 0.1 ± 0.02 ab | 0.101 ± 0.008 ab | 0.08 ± 0.02 b | 0.081 ± 0.005 b | 0.09 ± 0.01 b | 0.095 ± 0.009 ab | 0.12 ± 0.01 ab | n.d. ab | 0.13 ± 0.03 a | 0.1 ± 0.01 ab |
| ^Y^ indicates that compounds production was significantly affected by the factor yeast (pvalue < 0.05). *n.d.* indicates that the compound was not detected (below detection limits). | | | | | | | | | | |

| Table S3 Concentrations of trace aroma compounds (µg/L) found in the unfermented control and in the young and aged wines fermented by two selected *S. cerevisiae* yeasts, with and without PAF addition in must. Amounts of vitispirane and Riesling acetal are expressed in relative area since pure compounds were not available. | | | | | | | | | | |
| --- | --- | --- | --- | --- | --- | --- | --- | --- | --- | --- |
|  | MUST | | CLOS | | | | IONYS | | | |
|  | young+PAF | aged+PAF | young-PAF | young+PAF | aged-PAF | aged+PAF | young-PAF | young+PAF | aged-PAF | aged+PAF |
| **Esters** |  |  |  |  |  |  |  |  |  |  |
| isobutyl acetate ^Y^ | n.d. | n.d. | 6.3 ± 0.7 | 8 ± 0.4 | 33 ± 5 | 33 ± 4 | 58 ± 20 | 76 ± 20 | 18 ± 6 | 26 ± 9 |
| b-phenylethyl acetate ^YTP^ | n.d. | n.d. | 32 ± 4 | 81 ± 20 | 23 ± 4 | 51 ± 9 | 886 ± 100 | 1509 ± 30 | 315 ± 40 | 585 ± 100 |
| ethyl isobutyrate ^YT^ | n.d. | n.d. | 6 ± 2 | 6.9 ± 0.2 | 383 ± 50 | 432 ± 70 | 8 ± 3 | 5 ± 2 | 372 ± 90 | 237 ± 30 |
| ethyl isovalerate ^YTP^ | n.d. | n.d. | 0.42 ± 0.08 | 0.83 ± 0.08 | 29 ± 4 | 41 ± 20 | 0.51 ± 0.01 | 0.7 ± 0.2 | 54 ± 9 | 58 ± 20 |
| ethyl 2-methylbutyrate ^YT^ | n.d. | n.d. | 0.72 ± 0.05 | 0.56 ± 0.04 | 36 ± 7 | 60 ± 10 | 0.7 ± 0.2 | 0.8 ± 0.2 | 68 ± 10 | 49 ± 3 |
| ethyl leucate ^YTP^ | n.d. | n.d. | n.d. | 10 ± 4 | 46 ± 10 | 73 ± 20 | n.d. | 28 ± 8 | 36 ± 10 | 139 ± 9 |
| ethyl dihydrocinnamate ^YP^ | n.d. | n.d. | n.d. | 0.021 ± 0.003 | n.d. | 0.020 ± 0.003 | n.d. | 0.058 ± 0.009 | n.d. | 0.0633 ± 0.004 |
| **Lactones** |  |  |  |  |  |  |  |  |  |  |
| g-octalactone ^YP^ | n.d. | n.d. | 0.4 ± 0.06 | 0.29 ± 0.09 | 0.37 ± 0.04 | 0.3 ± 0.1 | 1.22 ± 0.02 | 2 ± 1 | 1.1 ± 0.2 | 2.5 ± 0.6 |
| g-nonalactone ^YTP^ | n.d. | n.d. | 0.68 ± 0.02 | 2 ± 0.07 | 1 ± 0.1 | 2.6 ± 0.2 | 0.42 ± 0.07 | 2.3 ± 0.4 | 0.69 ± 0.04 | 2.86 ± 0.09 |
| g-decalactone | n.d. | n.d. | 1.4 ± 0.2 | 0.9 ± 0.1 | 1.43 ± 0.05 | 0.8 ± 0.3 | 1.6 ± 0.5 | 1.9 ± 0.1 | 11 ± 20 | 2 ± 0.3 |
| massoia lactone ^YTP^ | 0.74 ± 0.09 | 2.2 ± 0.7 | n.d. | 0.57 ± 0.05 | n.d. | 0.14 ± 0.02 | n.d. | 0.6 ± 0.2 | n.d. | 0.129 ± 0.003 |
| **Nor-isoprenoids** |  |  |  |  |  |  |  |  |  |  |
| b-damascenone ^YTP^ | 0.4 ± 0.2 | 8 ± 2 | n.d. | 2.8 ± 0.7 | n.d. | 8.3 ± 0.7 | n.d. | 4.3 ± 0.7 | n.d. | 9.1 ± 0.9 |
| b-ionone ^P^ | n.d. | n.d. | n.d. | 0.21 ± 0.05 | n.d. | n.d. | n.d. | 0.27 ± 0.23 | n.d. | n.d. |
| TDN ^YTP^ | n.d. | 156 ± 40 | n.d. | 2.6 ± 0.2 | n.d. | 132 ± 10 | n.d. | 2 ± 1 | n.d. | 105 ± 10 |
| vitispirane ^YTP^ | n.d. | 0.55 ± 0.08 | n.d. | n.d. | n.d. | 0.46 ± 0.05 | n.d. | n.d. | n.d. | 0.39 ± 0.02 |
| Riesling acetal ^YTP^ | n.d. | 0.22 ± 0.02 | n.d. | n.d. | n.d. | 0.17 ± 0.03 | n.d. | n.d. | n.d. | 0.21 ± 0.02 |
| **Terpenes** |  |  |  |  |  |  |  |  |  |  |
| linalool ^YTP^ | 0.977 ± 0.005 | n.d. | 2.5 ± 0.1 | 4 ± 0.3 | n.d. | 0.52 ± 0.08 | 77 ± 20 | 22 ± 6 | 2 ± 1 | 1.8 ± 0.7 |
| linalool oxide ^YTP^ | n.d. | 13 ± 4 | n.d. | 0.5 ± 0.4 | n.d. | 8 ± 1 | n.d. | 0.39 ± 0.05 | 2.3 ± 0.4 | 6.2 ± 0.2 |
| b-citronellol ^YTP^ | n.d. | n.d. | 1.36 ± 0.06 | 2.3 ± 0.3 | n.d. | 0.5 ± 0.1 | 2 ± 0.2 | 4 ± 1 | 0.1 ± 0.1 | 0.6 ± 0.3 |
| geraniol ^YTP^ | n.d. | n.d. | 4.7 ± 0.3 | 6.2 ± 0.5 | n.d. | n.d. | 47 ± 7 | 21 ± 2 | n.d. | n.d. |
| nerol ^YTP^ | n.d. | n.d. | 0.92 ± 0.04 | 0.9 ± 0.1 | n.d. | n.d. | 3.7 ± 0.9 | 0.97 ± 0.08 | n.d. | n.d. |
| **Vanillin derivatives** |  |  |  |  |  |  |  |  |  |  |
| vanillin ^YP^ | 6.90 ± 0.26 | 22 ± 10 | n.d. | 5 ± 2 | n.d. | 21 ± 1 | n.d. | 6 ± 3 | n.d. | 20 ± 0.7 |
| acetovanillone ^YP^ | 3.9 ± 0.9 | 5.8 ± 0.7 | n.d. | 36 ± 4 | n.d. | 36.5 ± 0.2 | n.d. | 37 ± 3 | n.d. | 38 ± 2 |
| syringaldehyde ^YTP^ | 236 ± 5 | 267 ± 60 | 0.1 ± 0.3 | 17 ± 20 | n.d. | 57 ± 10 | n.d. | 11 ± 9 | n.d. | 57 ± 30 |
| **Volatile phenols** |  |  |  |  |  |  |  |  |  |  |
| syringol ^YTP^ | 3.5 ± 0.2 | 143 ± 20 | n.d. | 4 ± 1 | n.d. | 73 ± 10 | n.d. | 4 ± 2 | n.d. | 57 ± 9 |
| guaiacol ^YTP^ | n.d. | 7 ± 1 | n.d. | 0.8 ± 0.4 | n.d. | 4 ± 0.6 | n.d. | 1.1 ± 0.9 | n.d. | 4.4 ± 0.2 |
| 4-ethylguaiacol | n.d. | n.d. | n.d. | 0.108 ± 0.009 | n.d. | n.d. | n.d. | 0.37 ± 0.08 | n.d. | n.d. |
| 4-ethylphenol ^YTP^ | n.d. | 0.28 ± 0.08 | n.d. | 0.17 ± 0.04 | 0.1 ± 0.1 | 0.45 ± 0.06 | n.d. | 0.16 ± 0.01 | n.d. | 0.327 ± 0.004 |
| 4-vinylguaiacol ^P^ | 9 ± 3 | 21 ± 2 | n.d. | 28 ± 7 | n.d. | 19 ± 5 | n.d. | 40 ± 30 | n.d. | 50 ± 20 |
| 4-vinylphenol ^YP^ | 6.5 ± 0.6 | 172 ± 10 | n.d. | 163 ± 50 | n.d. | 137 ± 30 | n.d. | 280 ± 90 | n.d. | 194 ± 40 |
| eugenol ^YP^ | n.d. | n.d. | n.d. | 0.42 ± 0.05 | n.d. | 0.57 ± 0.01 | n.d. | 0.53 ± 0.07 | n.d. | 0.54 ± 0.06 |
| methoxyeugenol ^YTP^ | 0.6 ± 0.1 | 5 ± 1 | n.d. | 1.2 ± 0.3 | n.d. | 6.4 ± 0.3 | n.d. | 1.1 ± 0.3 | n.d. | 5.2 ± 0.6 |
| trans-isoeugenol ^TP^ | 0.5 ± 0.2 | 0.23 ± 0.08 | n.d. | 0.7 ± 0.1 | n.d. | 0.58 ± 0.07 | n.d. | 0.8 ± 0.2 | n.d. | 0.7 ± 0.2 |
| p-propylguaiacol | n.d. | n.d. | n.d. | 0.1 ± 0.02 | n.d. | 0.093 ± 0.007 | n.d. | 0.101 ± 0.008 | n.d. | 0.11 ± 0.02 |
| ^Y,^ ^T^ and ^P^ indicates that the compound was significantly affected by yeasts, time and/or PAF addition, respectively pvalue < 0.05). *n.d*., indicates that the compound was not detected or below detection limits. | | | | | | | | | | |

| Table S4.- Results of the 3-way ANOVA for the factors yeast, aging time and PAF addition on trace compounds concentrations in the fermentations realized with and without PAF with 2 *S. cerevisiae* strains. | | | | | | | |
| --- | --- | --- | --- | --- | --- | --- | --- |
| Compounds | pvalue (yeast) | pvalue (time) | pvalue (PAF) | pvalue (yeast x time) | pvalue (yeast x PAF) | pvalue (time x PAF) | pvalue (yeast x time x PAF) |
| isobutyl acetate | **2,19E-06** | 6,48E-02 | 1,44E-01 | **1,24E-06** | 1,97E-01 | 5,17E-01 | 6,40E-01 |
| b-phenylethyl acetate | **7,41E-18** | **4,99E-12** | **2,54E-09** | **4,94E-12** | **3,56E-08** | **5,75E-04** | **1,61E-03** |
| ethyl isobutyrate | **8,38E-07** | **5,30E-13** | 2,37E-01 | **1,33E-06** | **1,72E-02** | 2,55E-01 | **2,00E-02** |
| ethyl isovalerate | **9,73E-08** | **1,50E-14** | **3,10E-03** | **1,42E-07** | **2,18E-04** | **4,43E-03** | **2,41E-04** |
| ethyl 2-methylbutyrate | **3,44E-04** | **6,21E-09** | 7,63E-01 | **4,34E-04** | 4,05E-01 | 7,69E-01 | 3,91E-01 |
| ethyl leucate | **1,01E-07** | **7,93E-12** | **2,36E-09** | **1,15E-05** | **9,82E-06** | **1,15E-05** | **1,69E-03** |
| ethyl dihydrocinnamate | **4.91E-10** | 2.08E-01 | **4.31E-11** | **1.87E-02** | **1.13E-08** | 7.69E-01 | 5.44E-01 |
| g-octalactone | **7,44E-08** | 4,30E-01 | **9,29E-03** | 6,48E-01 | **2,90E-03** | 1,81E-01 | 2,51E-01 |
| g-nonalactone | **7,19E-13** | **3,43E-06** | **1,47E-16** | **4,76E-02** | **3,00E-04** | **1,60E-02** | 9,18E-01 |
| g-decalactone | 3,07E-01 | 3,37E-01 | 2,89E-01 | 5,33E-01 | 4,06E-01 | 3,04E-01 | 3,17E-01 |
| massoia lactone | **3,99E-10** | 8,09E-01 | **9,57E-05** | **2,57E-07** | 9,03E-01 | **5,73E-03** | 8,30E-01 |
| b-damascenone | **1,26E-03** | **3,74E-11** | **4,58E-15** | **1,25E-06** | **3,41E-02** | **8,54E-09** | 4,67E-01 |
| b-Ionone | 6.62E-02 | 9.35E-01 | **1.86E-02** | 2.69E-01 | 4.88E-01 | 9.33E-02 | 4.08E-01 |
| TDN | **1,06E-06** | **1,32E-12** | **8,87E-11** | **7,70E-07** | 1,48E-01 | **1,71E-10** | 1,62E-01 |
| vitispirane | **1,07E-09** | **3,18E-16** | **4,35E-14** | **1,07E-09** | 1,11E-01 | **4,35E-14** | 1,11E-01 |
| Riesling acetal | **1,60E-07** | **4,94E-15** | **3,53E-13** | **1,60E-07** | 1,43E-01 | **3,53E-13** | 1,43E-01 |
| linalool | **1,57E-08** | **1,48E-08** | **3,40E-05** | **5,12E-08** | **1,44E-05** | **3,32E-05** | **2,19E-05** |
| linalool oxide | **9,75E-06** | **4,28E-10** | **1,74E-06** | **4,66E-06** | **3,60E-02** | **1,33E-05** | **4,91E-02** |
| b-citronellol | **1,51E-05** | **2,48E-09** | **7,45E-05** | **3,26E-04** | 3,32E-01 | 6,54E-02 | 4,23E-01 |
| geraniol | **1,04E-11** | **2,31E-13** | **5,76E-06** | **1,04E-11** | **1,24E-06** | **5,76E-06** | **1,24E-06** |
| nerol | **1,84E-06** | **3,15E-10** | **2,25E-05** | **1,84E-06** | **2,42E-05** | **2,25E-05** | **2,42E-05** |
| vanillin | **1,10E-03** | **3,37E-06** | **1,35E-08** | 2,26E-01 | 9,93E-01 | **3,49E-05** | 6,32E-01 |
| acetovanillone | **4,08E-10** | 6,19E-01 | **1,85E-20** | 6,71E-01 | 4,34E-01 | 8,97E-01 | 9,00E-01 |
| syringaldehyde | **2,67E-14** | **3,70E-03** | **1,68E-04** | 8,70E-01 | 8,58E-01 | **9,28E-03** | 8,39E-01 |
| syringol | **6,92E-11** | **9,74E-13** | **4,09E-10** | **1,05E-10** | 2,01E-01 | **3,17E-09** | 1,72E-01 |
| guaiacol | **2,27E-08** | **1,25E-11** | **2,41E-11** | **1,55E-09** | 3,94E-01 | **3,02E-08** | 8,89E-01 |
| 4-ethylguaiacol | **7,94E-06** | **1,46E-08** | **4,45E-09** | **7,94E-06** | **2,00E-05** | **4,45E-09** | **2,00E-05** |
| 4-ethylphenol | **1,39E-08** | **7,46E-09** | **4,06E-16** | **3,89E-04** | **6,73E-03** | **2,24E-09** | **1,11E-02** |
| 4-vinylguaiacol | 1,43E-01 | 6,68E-01 | **3,10E-06** | 4,68E-01 | 5,39E-02 | 9,39E-01 | 3,72E-01 |
| 4-vinylphenol | **3,58E-02** | 9,82E-01 | **2,60E-10** | **5,55E-04** | **1,14E-02** | 8,66E-02 | 3,42E-01 |
| eugenol | **1,62E-10** | **1,38E-02** | **1,58E-18** | **3,39E-02** | 1,54E-01 | **8,60E-03** | **1,66E-02** |
| methoxyeugenol | **5,83E-05** | **3,66E-13** | **1,41E-14** | **7,04E-05** | 6,53E-02 | **1,26E-11** | 1,12E-01 |
| trans-isoeugenol | 5,40E-01 | 1,14E-01 | **1,88E-11** | 1,41E-01 | 2,97E-01 | 4,35E-01 | 4,74E-01 |
| p-propylguaiacol | **1,59E-07** | 8,66E-01 | **9,79E-15** | 5,28E-01 | **5,12E-03** | 8,55E-01 | 2,66E-01 |
| pvalues in bold are inferior to 0.05. | | | | | | | |

| Table S5.- Concentrations of trace aroma compounds (µg/L) found in wines fermented by 10 S. cerevisiae yeasts with PAF (mean ± standard deviation) after accelerated aging. Letters indicates Tukey test results. Amounts of vitispirane and Riesling acetal are expressed in relative area since pure compounds were not available. | | | | | | | | | | |
| --- | --- | --- | --- | --- | --- | --- | --- | --- | --- | --- |
|  | **CLOS** | **IONYS** | **71B** | **BDX** | **D254** | **D80** | **HPS** | **OKAY** | **PERSY** | **RHONE** |
| **Esters** |  |  |  |  |  |  |  |  |  |  |
| isobutyl acetate ^Y^ | 33 ± 4 bcd | 26 ± 9 cd | 21 ± 2 d | 43 ± 3 abcd | 51 ± 10 ab | 65 ± 20 a | 48 ± 9 abc | 25 ± 2 d | 25 ± 6 d | 55 ± 4 ab |
| b-phenylethyl acetate ^Y^ | 51 ± 9 b | 585 ± 100 a | 137 ± 30 b | 91 ± 6 b | 53 ± 20 b | 109 ± 60 b | 64 ± 20 b | 110 ± 20 b | 137 ± 20 b | 85 ± 10 b |
| ethyl isobutyrate ^Y^ | 432 ± 70 abc | 237 ± 30 bc | 122 ± 10 c | 282 ± 20 bc | 531 ± 90 ab | 775 ± 300 a | 518 ± 100 ab | 169 ± 50 bc | 190 ± 50 bc | 468 ± 70 abc |
| ethyl 2-methylbutyrate ^Y^ | 41 ± 20 abc | 58 ± 20 a | 16 ± 0.2 c | 25 ± 5 bc | 52 ± 7 ab | 53 ± 9 ab | 48 ± 7 ab | 29 ± 5 abc | 23 ± 3 bc | 45 ± 8 abc |
| ethyl isovalerate ^Y^ | 60 ± 10 abcd | 49 ± 3 bcde | 23 ± 2 e | 38 ± 1 cde | 64 ± 20 abc | 85 ± 20 a | 64 ± 10 abc | 45 ± 5 bcde | 33 ± 3 de | 69 ± 9 ab |
| ethyl leucate | 73 ± 20 | 139 ± 9 | 69 ± 2 | 121 ± 3 | 73 ± 20 | 118 ± 40 | 120 ± 60 | 70 ± 3 | 115 ± 10 | 124 ± 20 |
| ethyl dihydrocinnamate ^Y^ | 0.020 ± 0.002 c | 0.063 ± 0.004 a | 0.0220 ± 0.001 bc | 0.020 ± 0.002 c | 0.020 ± 0.001 bc | 0.036 ± 0.009 b | 0.028 ± 0.006 bc | 0.053 ± 0.003 a | 0.027 ± 0.004 bc | 0.032 ± 0.007 bc |
| **Lactones** |  |  |  |  |  |  |  |  |  |  |
| g-octalactone ^Y^ | 0.3 ± 0.1 c | 2.5 ± 0.6 a | 0.54 ± 0.04 c | 0.63 ± 0.05 c | 0.7 ± 0.3 c | 0.8 ± 0.8 c | 0.9 ± 0.4 c | 2.1 ± 0.3 ab | 1.3 ± 0.3 bc | 0.43 ± 0.07 c |
| g-nonalactone | 2.6 ± 0.2 | 2.86 ± 0.09 | 2.6 ± 0.1 | 2.5 ± 0.3 | 2.7 ± 0.4 | 3 ± 0.1 | 2.7 ± 0.3 | 2.68 ± 0.02 | 2.9 ± 0.3 | 2.8 ± 0.1 |
| g-decalactone ^Y^ | 0.8 ± 0.3 bc | 2 ± 0.3 a | 0.94 ± 0.01 bc | 0.67 ± 0.03 c | 0.91 ± 0.06 bc | 0.88 ± 0.09 bc | 0.92 ± 0.06 bc | 0.93 ± 0.04 bc | 1 ± 0.1 bc | 1.12 ± 0.02 b |
| massoia lactone | 0.14 ± 0.02 | 0.129 ± 0.003 | 0.1 ± 0.01 | 0.13 ± 0.02 | 0.14 ± 0.04 | 0.11 ± 0.06 | 0.15 ± 0.02 | 0.15 ± 0.008 | 0.138 ± 0.006 | 0.15 ± 0.01 |
| **Nor-isoprenoids** |  |  |  |  |  |  |  |  |  |  |
| b-damascenone | 8.3 ± 0.7 | 9.1 ± 0.9 | 7.72 ± 0.06 | 7.5 ± 0.4 | 8 ± 1 | 9.1 ± 0.7 | 8 ± 1 | 8.1 ± 0.4 | 8.8 ± 0.1 | 9.1 ± 0.3 |
| TDN ^Y^ | 132 ± 10 a | 105 ± 10 abc | 55 ± 2 e | 96 ± 8 bcd | 108 ± 20 abc | 130 ± 10 a | 113 ± 9 abc | 71 ± 5 de | 87 ± 6 cd | 125 ± 9 ab |
| vitispirane ^Y^ | 0.46 ± 0.05 ab | 0.39 ± 0.02 abcd | 0.277 ± 0.008 e | 0.36 ± 0.02 cde | 0.43 ± 0.07 abcd | 0.49 ± 0.03 a | 0.45 ± 0.04 abc | 0.34 ± 0.03 de | 0.38 ± 0.02 bcd | 0.49 ± 0.02 a |
| Riesling acetal ^Y^ | 0.17 ± 0.03 ab | 0.21 ± 0.02 ab | 0.166 ± 0.002 b | 0.17 ± 0.02 ab | 0.19 ± 0.03 ab | 0.22 ± 0.02 a | 0.2 ± 0.02 ab | 0.19 ± 0.02 ab | 0.2 ± 0.01 ab | 0.212 ± 0.009 ab |
| **Terpenes** |  |  |  |  |  |  |  |  |  |  |
| linalool ^Y^ | 0.52 ± 0.08 c | 1.8 ± 0.7 a | 1.35 ± 0.04 ab | 1 ± 0.2 abc | 0.54 ± 0.08 c | 0.7 ± 0.2 bc | 0.48 ± 0.03 c | 1 ± 0.1 abc | 1 ± 0.2 abc | 0.46 ± 0.02 c |
| linalool oxide ^Y^ | 8 ± 1 ab | 6.2 ± 0.2 abcd | 4.18 ± 0.08 e | 5.6 ± 0.3 cde | 7 ± 1 abcd | 7.4 ± 0.4 abc | 7 ± 1 abcd | 5.4 ± 0.4 de | 5.9 ± 0.4 bcde | 8 ± 0.3 a |
| b-citronellol | 0.5 ± 0.1 | 0.6 ± 0.3 | 0.96 ± 0.05 | 0.6 ± 0.1 | 0.5 ± 0.2 | 0.9 ± 0.3 | 0.6 ± 0.2 | 1 ± 0.1 | 0.5 ± 0.1 | 0.7 ± 0.2 |
| **Vanillin derivatives** |  |  |  |  |  |  |  |  |  |  |
| vanillin | 21 ± 1 bcd | 20 ± 0.7 cd | 26 ± 2 ab | 28 ± 1 a | 23.6 ± 0.7 abc | 23 ± 1 abcd | 22 ± 1 bcd | 18 ± 3 d | 25 ± 4 abc | 19.5 ± 0.8 cd |
| acetovanillone | 36.5 ± 0.2 | 38 ± 2 | 38 ± 1 | 40 ± 2 | 37 ± 2 | 39 ± 4 | 35 ± 2 | 38 ± 2 | 38 ± 1 | 36 ± 2 |
| syringaldehyde | 57 ± 10 | 57 ± 30 | 113 ± 20 | 97 ± 30 | 75 ± 10 | 52 ± 20 | 63 ± 10 | 105.4 ± 0.8 | 99 ± 50 | 72 ± 10 |
| **Volatile phenols** |  |  |  |  |  |  |  |  |  |  |
| syringol | 73 ± 10 | 57 ± 9 | 81.8 ± 0.2 | 79 ± 4 | 81 ± 10 | 71 ± 20 | 74 ± 20 | 80 ± 8 | 78 ± 10 | 55 ± 3 |
| guaiacol ^Y^ | 4 ± 0.6 bc | 4.4 ± 0.2 bc | 6.59 ± 0.07 a | 5 ± 1 ab | 4.9 ± 0.2 ab | 4.6 ± 0.8 bc | 4.8 ± 0.6 b | 6 ± 1 ab | 5 ± 0.6 ab | 3 ± 0.2 c |
| 4-ethylphenol ^Y^ | 0.45 ± 0.06 a | 0.327 ± 0.004 b | 0.258 ± 0.002 b | 0.3 ± 0.02 b | 0.43 ± 0.05 a | 0.51 ± 0.05 a | 0.47 ± 0.02 a | 0.27 ± 0.04 b | 0.31 ± 0.01 b | 0.48 ± 0.03 a |
| 4-vinylguaicol ^Y^ | 19 ± 5 c | 50 ± 20 ab | 29 ± 1 bc | 20 ± 6 c | 17 ± 4 c | 24 ± 5 c | 17 ± 4 c | 53 ± 20 a | 22 ± 2 c | 19 ± 2 c |
| 4-vinylphenol | 137 ± 30 | 194 ± 40 | 170 ± 5 | 152 ± 30 | 135 ± 30 | 189 ± 40 | 133 ± 20 | 179 ± 40 | 148 ± 20 | 145 ± 10 |
| eugenol ^Y^ | 0.57 ± 0.01 abc | 0.54 ± 0.06 bc | 0.467 ± 0.006 c | 0.6 ± 0.06 ab | 0.65 ± 0.03 a | 0.62 ± 0.03 ab | 0.63 ± 0.01 ab | 0.63 ± 0.02 ab | 0.57 ± 0.04 abc | 0.59 ± 0.06 ab |
| methoxyeugenol ^Y^ | 6.4 ± 0.3 a | 5.2 ± 0.6 abc | 4.4 ± 0.4 c | 5.8 ± 0.3 ab | 5.9 ± 0.4 ab | 6.1 ± 0.4 ab | 5.6 ± 0.7 ab | 5 ± 0.2 bc | 5.2 ± 0.5 abc | 6 ± 0.2 ab |
| trans-isoeugenol | 0.58 ± 0.07 | 0.7 ± 0.2 | 0.6 ± 0.1 | 0.62 ± 0.02 | 0.6 ± 0.1 | 0.65 ± 0.04 | 0.55 ± 0.06 | 0.56 ± 0.02 | 0.61 ± 0.09 | 0.5 ± 0.03 |
| p-propylguaiacol | 0.093 ± 0.007 | 0.11 ± 0.02 | 0.12 ± 0.01 | 0.11 ± 0.02 | 0.09 ± 0.03 | 0.11 ± 0.02 | 0.093 ± 0.009 | 0.111 ± 0.005 | 0.132 ± 0.005 | 0.111 ± 0.005 |
| ^Y^ indicates that the compound was significantly affected by yeasts (pvalue < 0.05). *n.d.*, indicates that the compound was not detected or below detection limits. | | | | | | | | | | |

| Table S6.- Results of the 2-way ANOVA for the factors yeast and aging time and their interaction on trace compounds concentrations in the fermentations realized with PAF by 10 *S. cerevisiae* strains. | | | |
| --- | --- | --- | --- |
| Compounds | pvalue(yeast) | pvalue(time) | pvalue(time x yeast) |
| isobutyl acetate | **2,16E-08** | **4,99E-11** | **1,90E-11** |
| b-phenylethyl acetate | **2,88E-27** | **6,83E-13** | **6,92E-15** |
| ethyl isobutyrate | **1,36E-06** | **1,19E-18** | **1,82E-06** |
| ethyl isovalerate | **7,32E-09** | **5,17E-29** | **1,12E-08** |
| ethyl 2-methylbutyrate | **7,29E-05** | **7,53E-22** | **1,05E-04** |
| ethyl leucate | **5,17E-04** | **1,62E-19** | 5,94E-02 |
| ethyl dihydrocinnamate | **2.20E-16** | 6.02E-02 | **1.45E-02** |
| b-damascenone | **6,18E-07** | **8,71E-36** | **9,59E-03** |
| b-ionone | 3.55E-01 | **6.15E-03** | 1.28E-01 |
| TDN | **1,71E-11** | **3,07E-38** | **6,73E-11** |
| vitispirane | **4,18E-09** | **2,50E-42** | **4,18E-09** |
| Riesling acetal | **1,02E-02** | **8,74E-39** | **1,02E-02** |
| geraniol | **8,09E-30** | **2,03E-35** | **8,09E-30** |
| linalool | **6,38E-16** | **2,36E-15** | **1,20E-14** |
| linalool oxide | **1,25E-07** | **4,26E-37** | **4,28E-07** |
| b-citronellol | **7,56E-09** | **1,64E-23** | **1,93E-05** |
| nerol | **5,75E-03** | **5,78E-37** | **5,75E-03** |
| g-octalactone | **1,52E-09** | 7,13E-01 | 9,13E-01 |
| g-nonalactone | 2,52E-01 | **5,14E-13** | 8,57E-01 |
| g-decalactone | **4,64E-18** | 2,97E-01 | 8,99E-01 |
| massoia lactone | 3,01E-01 | **9,57E-23** | 6,90E-01 |
| vanillin | **7,37E-04** | **2,76E-24** | **3,36E-02** |
| acetovanillone | **2,75E-02** | 5,77E-01 | 8,61E-01 |
| syringaldehyde | **3,19E-05** | **1,70E-09** | 9,58E-01 |
| syringol | **4,35E-02** | **4,26E-31** | **3,05E-02** |
| guaiacol | **2,81E-04** | **3,80E-30** | **1,70E-05** |
| 4-ethylguaiacol | **1,23E-11** | **2,97E-18** | **1,23E-11** |
| 4-ethylphenol | **8,04E-12** | **2,21E-29** | **7,07E-11** |
| 4-vinylguaiacol | **2,56E-03** | 3,80E-01 | **1,44E-04** |
| 4-vinylphenol | **4,19E-06** | 3,87E-01 | **2,14E-04** |
| eugenol | **4,00E-04** | **2,02E-12** | 9,05E-02 |
| methoxyeugenol | **4,44E-03** | **1,38E-34** | **2,62E-03** |
| trans-isoeugenol | 5,30E-01 | **2,46E-02** | 6,18E-01 |
| p-propylguaiacol | **4,83E-04** | 5,32E-02 | 7,92E-01 |
| pvalues in bold are inferior to 0.05. | | | |
